# Visualization of cristae and mtDNA interactions via STED nanoscopy using a low saturation power probe

**DOI:** 10.1038/s41377-024-01463-9

**Published:** 2024-05-24

**Authors:** Wei Ren, Xichuan Ge, Meiqi Li, Jing Sun, Shiyi Li, Shu Gao, Chunyan Shan, Baoxiang Gao, Peng Xi

**Affiliations:** 1 Department of Biomedical Engineering, National Biomedical Imaging Center, College of Future Technology, Peking University, Beijing, 100871 China; 2 Key Laboratory of Analytical Science and Technology of Hebei Province, College of Chemistry and Material Science, Hebei University, Baoding, 071002 China; 3 School of Life Sciences, Peking University, Beijing, 100871 China; 4 National Center for Protein Sciences, Peking University, Beijing, 100871 China

**Keywords:** Super-resolution microscopy, Biophotonics

## Abstract

Mitochondria are crucial organelles closely associated with cellular metabolism and function. Mitochondrial DNA (mtDNA) encodes a variety of transcripts and proteins essential for cellular function. However, the interaction between the inner membrane (IM) and mtDNA remains elusive due to the limitations in spatiotemporal resolution offered by conventional microscopy and the absence of suitable in vivo probes specifically targeting the IM. Here, we have developed a novel fluorescence probe called HBmito Crimson, characterized by exceptional photostability, fluorogenicity within lipid membranes, and low saturation power. We successfully achieved over 500 frames of low-power stimulated emission depletion microscopy (STED) imaging to visualize the IM dynamics, with a spatial resolution of 40 nm. By utilizing dual-color imaging of the IM and mtDNA, it has been uncovered that mtDNA tends to habitat at mitochondrial tips or branch points, exhibiting an overall spatially uniform distribution. Notably, the dynamics of mitochondria are intricately associated with the positioning of mtDNA, and fusion consistently occurs in close proximity to mtDNA to minimize pressure during cristae remodeling. In healthy cells, >66% of the mitochondria are Class III (i.e., mitochondria >5 μm or with >12 cristae), while it dropped to <18% in ferroptosis. Mitochondrial dynamics, orchestrated by cristae remodeling, foster the even distribution of mtDNA. Conversely, in conditions of apoptosis and ferroptosis where the cristae structure is compromised, mtDNA distribution becomes irregular. These findings, achieved with unprecedented spatiotemporal resolution, reveal the intricate interplay between cristae and mtDNA and provide insights into the driving forces behind mtDNA distribution.

## Abstract (web summary)

HBmito Crimson has the ability to localize specifically on the inner mitochondrial membrane and emit light, while its superior photostability enables STED imaging.

## Graphical abstract

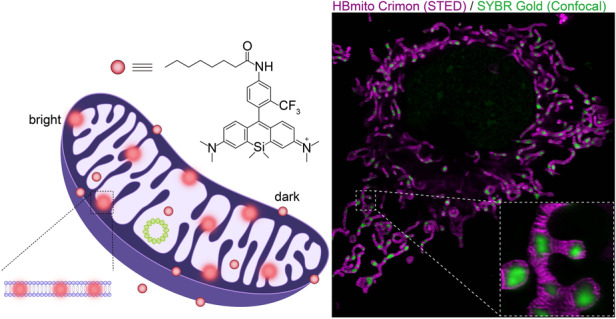

## Introduction

Mitochondria, as highly dynamic organelles, play a pivotal role in diverse biological processes, encompassing ATP production, iron-sulfur cluster biosynthesis, calcium homeostasis, and various cellular signaling pathways^1–5^. Structurally, as double-membrane organelles, Mitochondria are divided into four regions: outer membrane (OM), inner membrane (IM), intermembrane space, and matrix. The IM is further divided by the cristae junction (CJ) into the inner boundary membrane and the cristae, which are extensively folded invaginations enhancing the surface area^6,7^. Mitochondrial DNA (mtDNA) contains 37 genes and encodes 13 mitochondrial proteins^8^. mtDNA must be distributed throughout the mitochondrial network, as the OXPHOS complexes contain mtDNA-encoded subunits, which assemble in situ^8,9^. Movement of mtDNA located in the matrix compartment is limited due to the fact that mitochondrial cristae represent a substantial barrier to longitudinal free diffusion^10^. Therefore, mitochondrial fusion and fission involved in cristae remodeling are essential for the distribution and maintenance of mtDNA^11,12^. In this process, however, the mechanisms by which the dynamic properties of the cristae arrangement facilitate the distribution of mtDNA across the network have remained relatively underexplored, primarily due to the diffraction limit in optical microscopy and the lack of suitable in vivo markers for the mitochondrial IM.

Mitochondria, dynamic organelles sensitive to external pressures, typically range from 200 to 700 nm in diameter, with cristae spaced around 70 nm apart^13^. Therefore, it is impossible to observe cristae dynamics using conventional optical microscopes due to the ~200 nm spactial resolution limitation by diffraction. The advent of super-resolution imaging techniques has enabled the visualization of mitochondrial cristae (clusters)^14–20^. Among these techniques, structured illumination microscopy (SIM) provides a spatial resolution of ~90–120 nm^18,21–23^, which is insufficient for observing the dynamics of a single crista. Also, the results of SIM are heavily contingent on algorithmic reconstruction accompanied by an increase of possible artifacts when adjusting reconstruction parameters to yield clearer images, which requires considerable expertise to identify. Single-molecule localization (SML)-based methods such as PALM/STORM offer high enough spatial resolution, but their temporal resolution is limited (minutes per image), making it challenging to observe cristae dynamics effectively^17,24^. In contrast, stimulated emission depletion (STED) microscopy offers both spatial and temporal resolutions of ~50 nm and 1 s, respectively, making it the most promising tool for dynamic imaging of mitochondrial cristae at the single-crista level^16^.

Nevertheless, the application of STED in mitochondria has been constrained by probe photobleaching during time-lapse live-cell imaging. Also, the targeted accumulation of dyes on the mitochondrial IM is necessary to successful super-resolution imaging of mitochondrial cristae^25^. Moreover, STED requires a high-intensity depletion beam for resolution enhancement^26,27^, posing a challenge for dyes as they need to exhibit both high brightness and a high stimulated emission cross-section (i.e., low saturation power), while mitigating potential light-induced damage to mitochondria, which are sensitive to light stimulation. In previous studies, numerous novel probes were developed for mitochondrial STED dynamic imaging. MitoPB Yellow^15^ (λ_ex_ = 488 nm, λ_STED_ = 660 nm) and PK Mito Orange^13^ (λ_ex_ = 561 nm, λ_STED_ = 775 nm) exhibit outstanding photostability. However, their high-energy excitation wavelengths and low efficiency depletion hinder longer imaging and increase phototoxicity. MitoESq-635^19^ (λ_ex_ = 633 nm, λ_STED_ = 775 nm) enables STED dynamic imaging by effectively utilizing 775 nm pulsed laser depletion. Nonetheless, the inferior photostability of squaraine dye restricts its application for extended imaging periods.

In this study, we have engineered a novel fluorescent dye, HBmito Crimson, characterized by exceptional photostability, targeted accumulation on the mitochondrial IM, and emission of light only upon binding to the IM. Thanks to the overall redshift of spectrum and the emission spectral tailing effect leading to reduced saturation power, the efficiency of the 775 nm laser depletion has been significantly enhanced. By conducting comprehensive measurements of the chemical, biological, and optical properties of HBmito Crimson, STED imaging parameters can be optimized to better suit the dye’s characteristics. A spatial resolution of 40 nm and time-lapse imaging of over 500 frames were thus achieved on living cell mitochondria, enabling observation of mitochondrial network formation, fusion, fission, mtDNA duplication, apoptosis, and ferroptosis, facilitating recording of mitochondrial cristae dynamics at an unprecedented level of detail.

## Results

### Molecular design and optical properties of HBmito Crimson

**Fig. 1:**
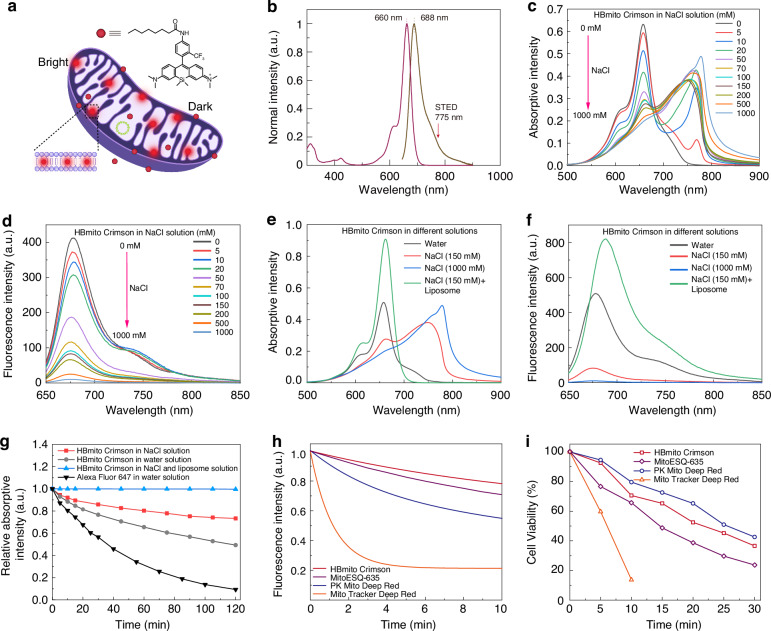
A lipid membrane light-up, highly photostable, lowly phototoxicity mitochondrial inner membrane probe. **a** Chemical structure of HBmito Crimson used for the specific labeling of the mitochondrial inner membrane. **b** The absorption and emission spectra of HBmito Crimson, which can be depleted using a 775-nm laser. **c**, **d** Absorption and fluorescence spectra of HBmito Crimson solution upon addition of different concentrations of NaCl (0-1000 mM). **e**, **f** Absorption and fluorescence spectra of HBmito Crimson solution in NaCl solution in the presence or absence of liposome. **g** The photostability of HBmito Crimson in different solutions and commercial Alexa Fluor 647 in water solution. **h** Photobleaching curve of HBmito Crimson, PK Mito Deep Red and Mito Tracker Deep Red in polymethyl methacrylate (PMMA). **i** Viability of HBmito Crimson, PK Mito Deep Red and Mito Tracker Deep Red-stained COS7 cells after Red light illumination (637 nm, 1.6 W/cm^2^)

Because of the negative membrane potential of mitochondria, Rhodamine cationic dyes have been extensively used as tools for staining mitochondria^28^. Si-Rhodamine compounds are far-red mitochondrial dyes with high fluorescence quantum yields, good photostability, and excellent membrane permeability. However, these rhodamine cationic dyes only accumulate in the mitochondrial matrix. Considering the fact that the tight bilayer of the mitochondrial IM is mainly composed of phospholipids, we hypothesized that highly selective mitochondrial IM dyes can be designed by introducing a lipophilic alkyl chain to Si-rhodamine dyes. Thus, we prepared HBmito Crimson by modifying Si-Rhodamine with lipophilic octanoate (Fig. 1a), in which HB represents both High-Brightness and He Bei, where the dye is invented.

In pure organic solvents or water, HBmito Crimson exhibited its longest absorption peak at ~660 nm and an emission peak at ~690 nm (Fig. 1b). Compared to the pure water solution, the absorption of HBmito Crimson in the PBS solution was red-shifted by ~100 nm and was accompanied by fluorescence quenching (Fig. S1). We speculated that this could be attributed to chloride ion-induced aggregation of the cationic rhodamine dye^29^. Furthermore, the aggregation behavior of HBmito Crimson was studied by ion-concentration-dependent UV-vis absorption and fluorescence spectroscopy (Fig. 1c, d). After adding chloride ions into a water solution of HBmito Crimson, a new absorption band, with the peak at 770 nm, appeared, indicating the aggregation of HBmito Crimson^30^, which was characterized by a red shift in the absorption spectrum and a decrease in fluorescence intensity at 660 nm. Increasing the chloride concentration to 1000 mM resulted in a continuous rise in the 770 nm peak, while the 680 nm peak decreased, concomitant with a decline in fluorescence intensity. Moreover, the addition of 1000 mM chloride ions led to complete fluorescence quenching. Additionally, dynamic light scattering (DLS) measurements showed that the aggregate sizes ranged from 50 nm to 700 nm upon the addition of chloride ions (Fig. S2).

Upon the addition of liposomes to a water solution containing 1000 mM NaCl, HBmito Crimson demonstrated a remarkable 80-fold fluorescence enhancement and a blue-shifted maximum absorption, accompanied by an increased extinction coefficient (Fig. 1e, f). Eukaryotic cells and their organelles (such as mitochondria), enveloped by lipid membranes comprised of lipid molecules organized into bilayers, can effectively simulate the behavior of HBmito Crimson in a real biological environment by adding liposomes. These observations strongly indicate that hydrophobic interactions drive HBmito Crimson to bind to lipid bilayers, effectively disrupting the ion-induced aggregation and resulting in enhanced fluorescence emission^31^. These results conclusively establish HBmito Crimson as a fluorogenic probe.

### Photostability and cytotoxicity of HBmito Crimson

To investigate the photostability of HBmito Crimson in different solutions, we monitored the changes in absorbance intensity over time during laser irradiation (Fig. 1g). Among the tested solutions, HBmito Crimson in the solution containing the liposomes exhibited the highest photostability, showing negligible photobleaching even after 2 h of exposure under 1 Watt 660-nm laser illumination. In contrast, significant photobleaching was observed when HBmito Crimson was in NaCl solution or water under the same conditions. This result demonstrated that the liposomes also play a crucial role in enhancing the photostability of HBmito Crimson in the liposome solution. The improved photostability of HBmito Crimson in the liposome solution could be attributed to the lipid double bonds acting as radical scavengers to protect HBmito Crimson from radical attack^32^. The photostability of HBmito Crimson was also compared with that of other mitochondrial dyes using hydrophobic polymethyl methacrylate (PMMA) films. Employing these hydrophobic PMMA films ensures uniform dispersion of probe molecules on the film surface^13^, thereby facilitating quantitative comparisons with other probes. HBmito Crimson, MitoESQ-635^19^, PK Mito Deep Red^33^, and Mito Tracker Deep Red were applied to these films, and time-lapse imaging was conducted under the same light intensity. Remarkably, HBmito Crimson demonstrated the highest level of photostability, with only 22% of the dye being photobleached after 10 min of irradiation with a 640 nm laser (Fig. 1h).

The cytotoxicity of HBmito Crimson was determined by evaluating cell viability using a standard MTT assay. HeLa cell viability was >95% after 24 h of incubation with 10 μM HBmito Crimson, indicating that HBmito Crimson demonstrated good biocompatibility (Fig. S3). To assess the phototoxicity of the probe, we compared HBmito Crimson with MitoESQ-635^19^, PK Mito Deep Red^33^ and Mito Tracker Deep Red (Fig. 1i). It is demonstrated that the phototoxicity of HBmito Crimson was similar to that of PK Mito Deep Red, lower that of MitoESQ-635 and significantly lower than that of Mito Tracker Deep Red. These results indicate that HBmito Crimson exhibits excellent photostability and low cytotoxicity, making it a favorable option for mitochondrial imaging applications.

HBmito Crimson has higher brightness compared to PK Mito Orange^13^, regardless of their respective optimal excitation bands (Fig. S4a, c) or the most commonly set excitation bands of commercial microscopes (Fig. S4b). To confirm the specificity of HBmito Crimson, we conducted imaging to assess co-localization with Mito Tracker Green (label mitochondria) and PK Mito Orange (label mitochondria)^13^. The analysis yielded high co-localization coefficients of 0.91 and 0.89, respectively. Furthermore, co-localization analysis with ER-KDEL (label endoplasmic reticulum) and Nile Red (label liposome) was performed, revealing co-localization coefficients of only 0.09 and 0.21. These results confirm that HBmito Crimson rarely labels other organelles and can specifically label mitochondria (Fig. S5).

### HBmito Crimson saturation power measurement

**Fig. 2:**
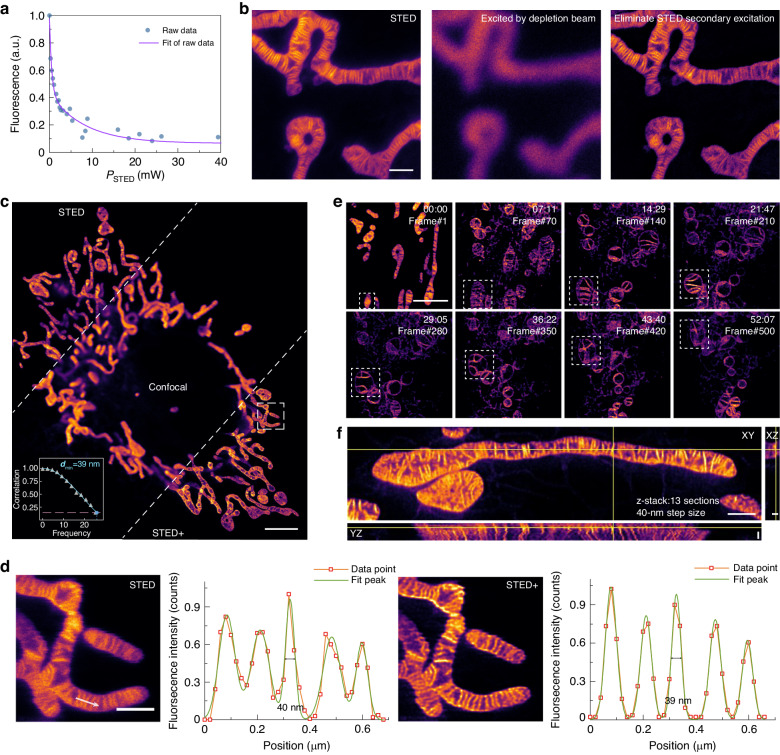
Multi-dimensional imaging of the mitochondrial inner membrane stained with HBmito Crimson. **a** The detected fluorescence signal of the HBmito Crimson solution dye pool on a coverslip as a function of the STED depletion beam intensity; the excitation beam was 660 nm. **b** Comparison of STED, only excited by depletion beam, and eliminates STED secondary excitation imaging results. **c** Comparison of confocal, STED, and STED+ (deconvolution with Huygens) imaging results in living COS7 cell mitochondria labeled with HBmito Crimson. The upper right corner shows the enlarged results for STED + . The lower left corner shows the Fourier ring correlation (FRC) calculation for STED + . **d** The enlarged STED and STED+ results and the fluorescence signal intensity profile corresponding to the white arrow. **e** A long period time-lapse STED imaging of mitochondria. White dashed boxes indicate the same mitochondrion. **f** 3D-STED imaging of cristae in COS7 cells labeled with HBmito Crimson. Scale bar in **b,** **c,** **d** and **e** are 1 μm, 5 μm, 1 μm and 3 μm. Scale bar in **f** is 1 μm for the XY view and 0.2 μm for the XZ and YZ view

In the STED imaging method, the saturation power is directly related to the resolution. Measuring the saturation power of the dye can facilitate the determination of the optimal laser power required for depletion and accurately set up the theoretical PSF formation in deconvolution. To accurately assess the dye saturation power, the phase distribution of the depletion laser in the commercial STED system is adjusted, converting it from a donut distribution to a gaussian distribution. HBmito Crimson exhibited a very low saturation power of 0.864 mW (*I*_s_) (Fig. 2a), which is impressively lower than the 3.069 mW saturation power for the classic STED probe ATTO 647N^19^. Based on the depletion beam power 34.7 mW (*I*_dep_) used in the time-lapse imaging, according to formula $\sqrt{1+{I}_{{\rm{dep}}}/{I}_{{\rm{s}}}}$, a 6-fold resolution enhancement can be achieved. In comparison, achieving a similar level of resolution with ATTO 647 N necessitates a depletion laser power input of 100 mW.

The stimulated emission cross-section is related to the emission spectrum of the dye^34,35^, and the tailing of the emission spectrum of HBmito Crimson results in lower saturation power. The results of STED and only excited by depletion beam (Gaussian distribution) were obtained by modifying the acquisition mode, with subtraction of the only excited by depletion beam image from STED image yielding the outcomes of eliminating STED secondary excitation^35^. Upon comparison, it was found that the impact of the secondary excitation is limited (Fig. 2b) and does not affect the resolution (Fig. S6). Thus, the enhancement in depletion efficiency due to the red shift of the HBmito Crimson spectrum is proved to be highly effective. Collectively, These data demonstrate that HBmito Crimson has extraordinary characteristics including high photostability, fluorescence quenching induced by chloride ions, fluorescence emission activated by lipid membranes, low saturation power, and localization within mitochondrial IM. These characteristics provide a solid foundation for conducting time-lapse STED imaging of the mitochondria cristae dynamics.

### Multi-dimensional imaging of the mitochondrial inner membrane

The advancements in STED technology have enabled visualization of the fine cristae structures in living cells, making it feasible to investigate the structure–function relationship of cristae in real time. HBmito Crimson, as a fluorogenic probe, facilitates wash-free STED experiments, as it is remarkably resistant to photobleaching and suitable for long-term STED imaging. Firstly, we implemented single-frame wash-free live-cell imaging using HBmito Crimson-labeled COS7 cells, and the results revealed that mitochondrial cristae were clearly resolved under STED and STED+ (Results was processed with the commercial deconvolution software Huygens (SVI, Netherlands) using default parameters set to “conservative” mode.) in comparison with confocal (Fig. 2c). The absolute resolution of STED and STED+ obtained by Fourier ring correlation (FRC) reached 40 nm and 39 nm^36^, respectively, and the full width at half maximum was fitted to 44 nm and 39 nm (Fig. 2d), respectively.

Subsequently, we used HBmito Crimson to perform low-power time-lapse STED imaging to visualize the dynamic changes in mitochondrial internal spatial structure. Under wash-free conditions, single crista was distinctly identified and their dynamics were monitored for >500 frames, for a total period of 52 min (Fig. 2e and Movie S1). Such a long imaging time demonstrates the excellent photostability of HBmito Crimson under STED imaging. Comparisons with time-lapse imaging results of mitochondrial IM probes reported in other references^13,15,19,37^ are shown in the Fig. S7 and Table S1. The combination of HBmito Crimson and STED technology enables the real-time observation of mitochondrial cristae dynamics with unparalleled resolution and stability. These advancements hold great promise for advancing our understanding of mitochondrial function and dynamics in living cells.

Cytoplasmic level of the divalent cation calcium (Ca^2+^) as an indicator of cell stress that can evaluate the impact of STED in live-cell imaging^38,39^. COS7 cells were initially incubated with the Ca^2+^ sensitive dye Fluo4, followed by staining with HBmito Crimson. In order to prevent Fluo4 from being bleached during the imaging process, we continuously imaged 10 frames of STED, and then took 1 frame of Fluo4 under confocal microscopy, and monitored the change of calcium signal during HBmito Crimson imaging. Monitoring the changes in calcium signals during the low-power STED imaging process, we observed that the fluctuation of calcium signals was slightly higher than that of confocal imaging within 12 min of imaging time. However, it was significantly lower than the positive control group experiment with the addition of an inducer of cell stress (Ionomycin, IO), indicating that time-lapse low-power STED imaging does not cause significant cell stress (Fig. S8).

The pursuit of 3D STED imaging in living cells presents challenges due to probe photobleaching and the rapid movement of mitochondria. Nevertheless, leveraging the exceptional photostability and brightness of HBmito Crimson, we successfully captured the 3D cristae structure of mitochondria in COS7 cells within 20.15 s (Fig. 2f). In the YZ orthogonal section, the mitochondrial cristae were observed to be arranged in a side-by-side fashion, while the XZ section revealed a hollow out region within the mitochondria.

These results demonstrate the immense potential of HBmito Crimson and STED technology in unraveling the intricate dynamics of mitochondrial cristae in living cells, opening up new avenues for comprehensive investigations into mitochondrial biology.

### Propensity of mtDNA distribution in the mitochondrial network

**Fig. 3:**
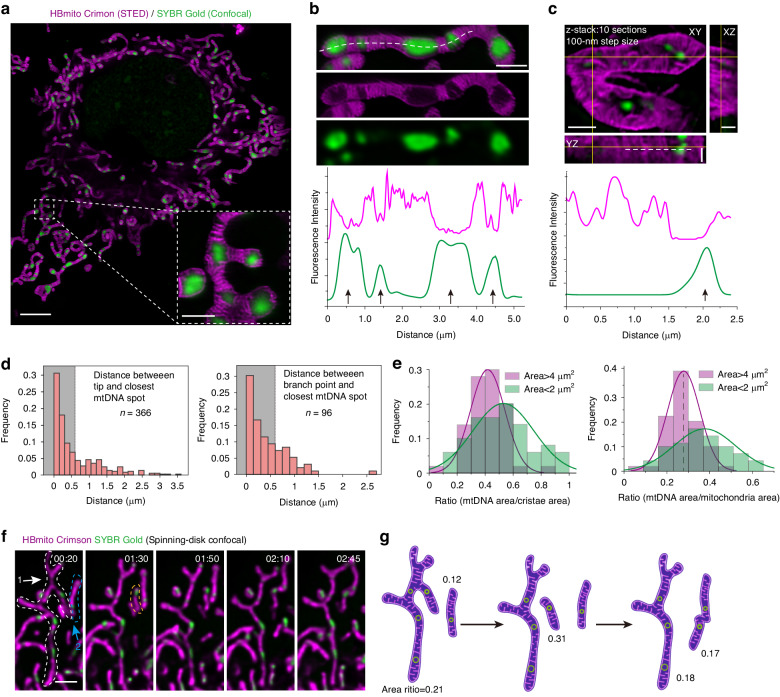
Distribution of cristae and mtDNA in mitochondria. **a** Dual-color live-cell imaging for mitochondria (STED) and mtDNA (confocal). **b** The pixel intensity of HBmito Crimson and SYBR Gold from a line scan drawn along the mitochondria (dashed line); arrows indicate mtDNA positions. **c** The pixel intensity of HBmito Crimson and SYBR Gold from a line scan drawn along the mitochondria. The white dashed line goes along the YZ direction. **d** Distance frequency distribution histogram from mitochondrial tips or branch points to the nearest mtDNA. The proportion of the gray part (distance < 0.6 µm) is 65.0% and 67.7%, respectively. **e** Frequency distribution histogram of the ratio of mtDNA area to cristae area or mitochondria area. **f** Small mitochondrial branches detach from the mitochondrial network and fuse with nearby mitochondria. White and blue dashed lines represent the mitochondrial network before fission and mitochondria before fusion, respectively. The yellow dotted line represents the small mitochondria after division. **g** Cartoon corresponding to (**f**). Scale bar in **a** is 5 μm and enlarged image in **a** is 1 μm. Scale bar in **b** and **f** are 1 μm and 2 μm. Scale bar in **c** is 0.5 μm for XY, XZ and YZ views

The exceptional stability exhibited by HBmito Crimson and the reliability of the live-cell STED imaging technique has established a robust method for studying how the dynamics of mitochondrial cristae membranes influence the distribution of mtDNA. We imaged the IM with HBmito Crimson labeling and mtDNA with SYBR™ Gold nucleic acid gel dye (Ex: 488 nm, Thermo Fisher, US, No. S11494) labeling (Fig. 3a). Due to the stability and spectrum constraints of the mtDNA labeling dye (which requires the same depletion laser for STED dual-color simultaneous imaging), STED was utilized for imaging IM, while confocal microscopy was employed for mtDNA imaging (Unless otherwise specified, all dual-color imaging methods are in this mode.). By performing dual-color imaging by 2D and 3D STED nanoscopy, we observed that mitochondrial cristae are often arranged in clusters, and the mtDNA generally occupies empty place in the matrix between cristae clusters (Fig. 3b, c). In comparison with references^10,14^, we were able to observe mitochondrial cristae at a resolution of 40 nm in living cells. A similar mtDNA localization was further observed under 3D SIM and 2D SIM microscopy (Figs. S9 and S10). The mtDNA labeled with SYBR Gold was further imaged by STED, and the size of mtDNA observed under STED was proved to be significantly smaller than attainable with conventional confocal microscopy, as shown in Fig. S11, and mtDNA clusters were clearly resolved under STED.

Additionally, our findings indicated that mtDNA exhibits a regular spatial arrangement within mitochondrial networks, with a preference for distribution at the tips and branch points (Fig. 3d). We then quantitated the propensity of mtDNA distribution and created a frequency distribution histogram by measuring the distances between the mitochondrial tips (*n* = 366) or mitochondrial branch points (*n* = 96) and the nearest mtDNA (Fig. 3d). Interestingly, these mtDNA near mitochondrial tips or branch points show an exponential distribution, suggesting that mtDNA tends to locate at mitochondrial tips or branch points of mitochondrial networks^40^ (Fig. 3d). Recent evidence showed that after mtDNA replication, ER-linked mitochondrial fission occurs between the replicated mtDNA^41^, which also indicated that mtDNA located at newly generated mitochondrial tips after fission. Due to constraints such as mtDNA volume, intra-mitochondrial protein content, and mitochondrial cristae, the mobility of mtDNA within mitochondria is limited^42^. Therefore, we assume that the distribution of mtDNA at the tips and branch points ensures the rapid and efficient allocation of mtDNA during mitochondrial fusion and fission. Moreover, through dynamic mtDNA positioning, cells can redistribute their energy-generating capacity to specific regions, which might be important for mitochondria adapt to increased energy demand or cellular stress. Subsequently, the spatial changes in mtDNA and mitochondrial cristae at the tips were dynamically observed, revealing a tendency for mitochondrial cristae to tether mtDNA, thereby preventing its movement (Fig. S12).

The difference in mtDNA distribution between small mitochondria and mitochondrial networks was examined by quantifying the ratio of mtDNA area to mitochondrial cristae area. Through this analysis, it was observed that the area ratio (mtDNA area/cristae area) of small mitochondria (0.524) is higher than that of mitochondrial networks or large mitochondria (0.421) (Fig. 3e). We hypothesize that large mitochondria with a low area ratio and dense cristae are used to produce ATP to maintain cellular function, and small mitochondria with a high area ratio can serve as the carriers of mtDNA to achieve even distribution of mtDNA through floating and fusion processes. Further investigation into the relationship across the entire mitochondrial area revealed that the area ratio (mtDNA area/mitochondrial area) of small mitochondria (0.377) remained higher than that of mitochondrial networks or large mitochondria (0.277) (Fig. 3e). This further illustrated that the area ratio is different in mitochondria of different sizes, and STED also facilitates the area ratio measurement with high accuracy. The hypothesis was validated by observing the detachment of small mitochondrial branches from the mitochondrial network and subsequent fusion with nearby mitochondria (spinning disk confocal microscopy, Fig. 3f, g and Movie S2). The area ratios of mitochondrial networks 1 and 2 were measured to be 0.21 and 0.12, respectively, as indicated by the white and blue dashed lines. The area ratio of mitochondrial network 2 fused with small mitochondria (area ratio 0.31, yellow dashed lines) reached 0.17, which was close to the average level, and the area ratio of mitochondrial network 1 after splitting was 0.18. A cartoon corresponding to this process is shown in Fig. 3g. According to the above results of different mitochondrial area ratios, it is demonstrated that small mitochondria can be separated from the mitochondrial network and transport mtDNA to other mitochondrial networks to balance the even distribution of mtDNA.

### Mitochondrial dynamics associated with mtDNA

The formation and maintenance of the mitochondrial network is critical to the performance of mitochondrial functions (such as respiratory capacity, material exchange, and mtDNA integrity, etc.)^43,44^. In comparison with other organelles (e.g., peroxisomes and lysosomes), mitochondria cannot be created de novo, and only rely on dynamic behaviors such as fission and fusion to generate daughter mitochondria or form networks. In addition, mtDNA must be replicated in order to be transmitted to daughter mitochondria.

**Fig. 4:**
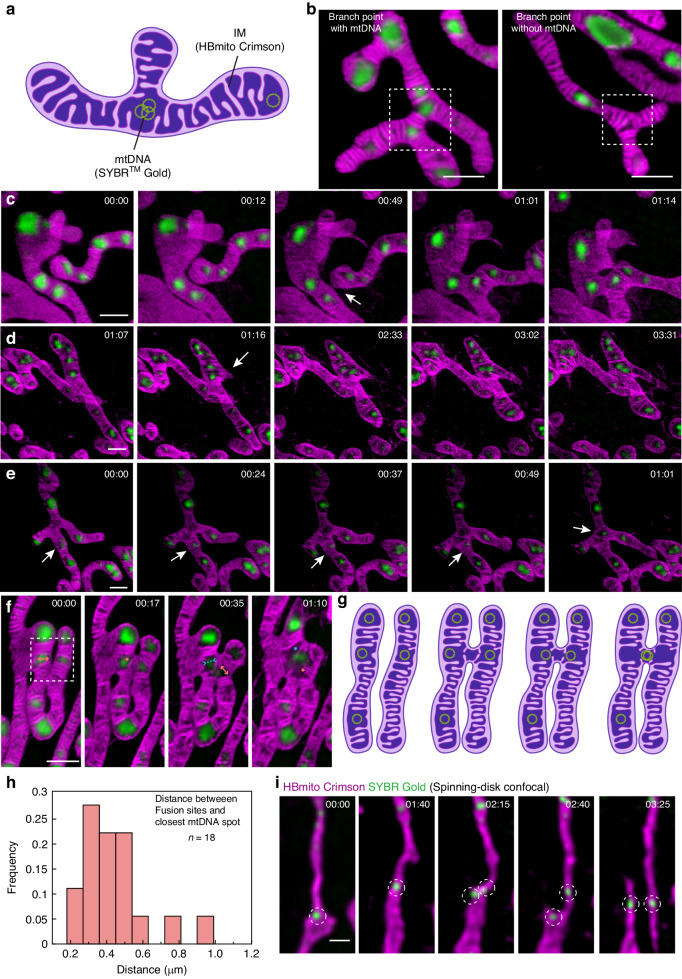
Mitochondrial dynamics associated with mtDNA. **a** Cartoon showing that mtDNA prefers to be distributed at the tips and branch points. **b** Presence (left) and absence (right) of mtDNA at branch points; the white box represents the branch point. **c** Branch point formation by mitochondrial fusion; white arrow indicates fusion sites. **d** Branch point formation by emersion of a new branch; white arrow indicates from where the new branch was generated. **e** The mtDNA on the mitochondrial branch moves to the branch point; white arrows indicate the movement of mtDNA. **f** Fusion process of mitochondria. Merging and splitting events of cristae are indicated by blue and orange asterisks, respectively, with bidirectional arrows facing inward (blue) and outward (orange) representing impending cristae merging and splitting events, respectively. **g** Cartoon showing the fusion process corresponding to the white box in (**f**). **h** Distance frequency distribution histogram. **i** mtDNA replication initiates mitochondrial division. The dotted line shows the replication of mtDNA. Scale bars are all 1 μm

The branch point is at the core of the mitochondrial network. Through dual-color imaging, we investigated the cristae arrangement and mtDNA distribution at the mitochondrial branch point and made a significant observation: regardless of the presence or absence of mtDNA at the branch point, the mitochondrial cristae were consistently oriented perpendicular to the extension direction of the branch. As a result, a unique trifurcated arrangement of cristae was formed near the branch point. From the results in Fig. 3d, it can be found that the probability of the presence of mtDNA at the branch point is 0.677. In the presence of mtDNA, the void matrixes (empty space in matrix) left by the cristae arrangement were filled; however, in the absence of mtDNA, a distinct space was left at the junction (Fig. 4a, b).

Branch point formation in mitochondrial networks can occur through two distinct processes, and their relationship with mtDNA distribution is a critical aspect to consider. The first mechanism involves mitochondrial fusion, where two individual mitochondria come together, and the site of fusion becomes a new branch point. This process is highlighted in Fig. 4c (Movie S3), where two mitochondria approach each other, and their respective mtDNA is in close proximity to the fusion site. After fusion, a new branch point is established, and since the fusion occurs near mtDNA, the mtDNA naturally resides at the branch point.

The second mechanism of branch point formation involves the emergence of a new branch from an existing mitochondrial tubule^43,45^. For the first time, we visualized the dynamics of cristae and mtDNA during this process using STED imaging, as illustrated in Fig. 4d (Movie S4). At the first, a new branch extends from a region where mtDNA is spatially dispersed. As the new branch grows in length and width, the mitochondrial cristae at the extension site undergo rapid remodeling to adapt to the new structure. As a result, the number of cristae at the newly formed branch point decreases, leading to a wider range of mtDNA activity, and the cristae at the new branch become relatively sparse. Reference^45^ was constrained by resolution limitations and consequently unable to observe cristae dynamics during mitochondrial tubule extension process (Fig. S13a, b).

In both cases of branch point formation, mtDNA is found in close proximity to the newly generated branch point. Due to the regularity of cristae arrangement at the branch point, void matrixes will be left when there is no mtDNA at the branch point. In order to efficiently utilize the limited space, mtDNA will actively move to the junction. Figure 4e (Movie S5) illustrates this process, where mtDNA initially resides at the mitochondrial branch but gradually moves towards the branch point, as indicated by the white arrows. When mtDNA is present at the branch point, void matrixes are formed between the cristae clusters. However, in the absence of mtDNA, these void matrixes gradually disappear.

The dynamic process of mitochondrial fusion plays a crucial role in the distribution of mtDNA within the mitochondrial network. We observed that during fusion, two parallel mitochondria come closer together, and the IM undergoes remodeling, followed by the transmission of mtDNA between them (Fig. 4f and Movie S6). This indicates that mitochondrial cristae facilitate the transmission of mtDNA between different mitochondria through a series of remodeling processes. A cartoon corresponding to this process is shown in Fig. 4g. Compared with other findings in the reference^46^, a clearer dynamic process of the cristae is outlined (Fig. S 13c, d).

In multiple experiments, a consistent observation emerged: fusion events consistently occurred in close proximity to mtDNA (Fig. 4h). By measuring the Euclidean distance between the fusion site and the nearest mtDNA, we obtained a distance frequency distribution histogram showing an exponential distribution. This implies that fusion sites are extremely close to mtDNA. Since the void matrixes between cristae clusters, where mtDNA is located, are less dense and the cristae arrangement is loose, we hypothesized that the pressure for cristae remodeling is reduced when fusion occurs near mtDNA.

Interestingly, one mitochondrion without mtDNA usually tends to fuse with mitochondrial network with more mtDNA (Fig. S14). By contrast, we also observed even or uneven dissemination of mtDNA during mitochondrial fission (Figs. S15, S16).

MtDNA replication can be synchronized with fission to ensure the correct segregation of genetic material in most instances^41,47^. We observed the entire process from mtDNA replication to mitochondrial division(Both IM and mtDNA imaging results were acquired by spinning disk confocal microscopy) in a representative time-lapse series (Fig. 4i and Movie S7). Initially, this long mitochondrion has only one mtDNA. At 02:15, a nascent mtDNA appeared near the original mtDNA, then fission took place between two mtDNA, and finally, the original mitochondria were divided into two daughter mitochondria, each with one mtDNA. Meanwhile, mtDNA was located at newly generated mitochondrial tips after fission. This observation directly demonstrates the spatial link between replicating mtDNA and fission sites, allowing each mitochondrion to obtain a copy of the genome following division and thereby dispersing mtDNA throughout the mitochondrial network.

Our study provides valuable insights into the dynamic relationship between mitochondrial membrane dynamics, cristae remodeling, and mtDNA distribution. By understanding these processes at unprecedented spatiotemporal resolution, we can further unravel the complexities of mitochondrial dynamics.

### Breakdown of cristae structure in apoptosis leads to mtDNA convergence

MtDNA-encoded proteins, such as components of the ATP synthase, play a direct role in the formation and composition of cristae structure. The ATP synthase is able to form dimers and oligomers, consisting of rows of dimers, which shape the mitochondrial IM and thus contribute to cristae formation^48^. Normal cristae structure ensures that mtDNA can be evenly distributed, since cristae can form partitioning structures to prevent the aggregation of mtDNA. On the contrary, once the cristae structure fails to contain mtDNA, the mitochondria will face disaster.

**Fig. 5:**
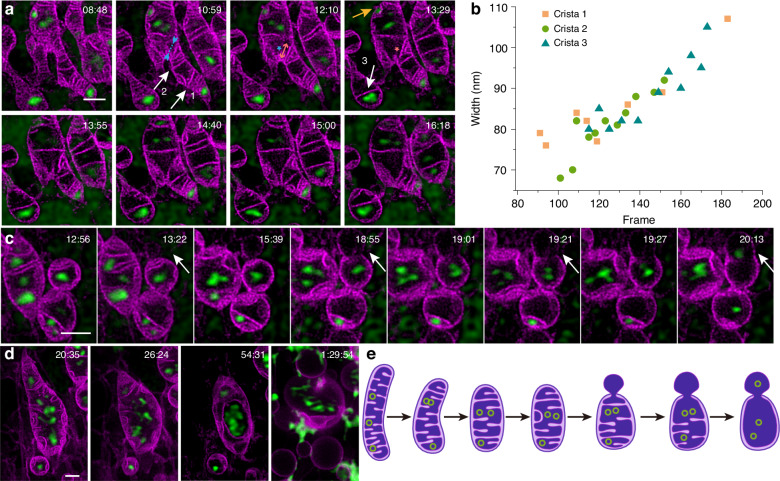
IM dynamics and mtDNA spatial changes during apoptosis. **a** Cristae remodeling and mtDNA convergence during early apoptosis. The yellow arrow indicates that cristae are attached to the inner boundary membrane in an arc. Merging and splitting events of cristae are indicated by blue and orange asterisks, respectively, with bidirectional arrows facing inward (blue) and outward (orange) representing impending cristae merging and splitting events, respectively. **b** Mitochondrial cristae gradually widened as the imaging frame number increased. The three curves (scatter plot) represent the three cristae indicated by white arrows 1, 2, and 3 in (**a**), respectively. **c**, **d** IM herniation and mtDNA leakage along with cristae remodeling; white arrows represents the manner of mitochondrial herniation. **e** Model of IM dynamics and mtDNA leakage during apoptosis. Scale bars are all 1 μm

During 8 min of low power time-lapse STED imaging, the mitochondrial cristae remained largely intact. However, significant changes in the shape of the mitochondrial IM were noticed as the time-lapse imaging progressed (Fig. 5a and Movie S8). To quantify these changes, three mitochondrial cristae (white arrows 1, 2, and 3) were selected, and their width was measured as the imaging frame number increased (Fig. 5b). The results showed that the mitochondrial cristae gradually widened during this process. This process was also accompanied by other cristae remodeling processes, including cristae merging and splitting and attachment to the inner boundary membrane in an arc (yellow arrow). Additionally, the range of motion of mtDNA gradually increased in conjunction with these changes.

As reported in previous studies^49,50^, the formation of higher-order oligomers of BAX/BAK creates lipid pores within the OM, resulting in IM herniation and mtDNA efflux in apoptosis. As the imaging time increased, the dynamic process of IM herniation and mtDNA leakage, alongside cristae remodeling, was observed (Fig. 5c, Movie S9, Fig. 5d, Movie S10, Fig. 5e). The herniated IM formed a barbell-shaped structure, and mtDNA was observed to move into the herniated IM (indicated by white arrows in Fig. 5c). During IM herniation, cristae within the mitochondria were pulled out, gradually disappeared (Fig. 5c). As the cristae within the mitochondria reduced, mtDNA was completely discharged from the tethering of mitochondrial cristae (Fig. 5c). Ultimately, the cristae structure and the distribution of mtDNA collapsed simultaneously. After apoptosis, the dissipation of membrane potential causes membrane potential-dependent SYBR Gold dye molecules to diffuse out of the mitochondria, resulting in the green signal observed in the background, which may originate from nuclear DNA or mtDNA released into the cytoplasm. Comparing with the reference^50^ which presented single-frame SIM results of mitochondrial herniation (Fig. S13e–g), our study captured richer information at higher resolution. 10 μM ABT-737(MCE, US, HY-50907) and 2 μM S63845(MCE, US, HY-100741) were administered to induce apoptosis, and the same results as above were observed (Fig. S17).

These observations provide important insights into the dynamic changes that occur during apoptosis, particularly regarding the role of mitochondrial cristae and mtDNA redistribution in this cellular process. Understanding these intricate mechanisms may offer valuable targets for therapeutic interventions aimed at controlling cell death in various physiological and pathological contexts.

### The spatial location of mitochondrial cristae and mtDNA during ferroptosis

Ferroptosis is an iron-dependent form of regulated necrosis in which intracellular reactive oxygen species (ROS) accumulation exceeds redox homeostasis that is maintained by glutathione (GSH) and phospholipid hydroperoxidase enzymes. The small-molecule compound erastin disrupts cellular redox homeostasis by inhibiting the cystine-glutamate antiporter, leading to depletion of cellular cysteine and glutathione^51,52^. Morphologically, ferroptotic cells exhibit mitochondrial ultrastructural changes such as decreased volume, disruption of the mitochondrial OM, and reduced mitochondrial cristae^53^. However, the manner by which the spatial conformations of cristae and mtDNA change under ferroptosis remains unexplored.

**Fig. 6:**
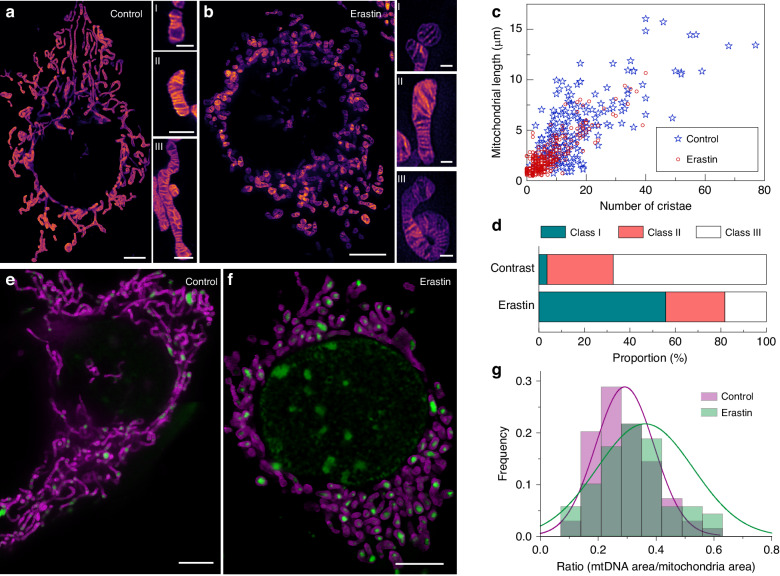
Spatial changes in mitochondrial cristae and mtDNA during ferroptosis by STED imaging. **a**, **b** Representative mitochondrial cristae images of control and erastin-treated cells. Mitochondria were classified into three categories based on length and number of cristae, as shown on the right side of the panel. **c** Plot of the mitochondrial length and number of cristae in control and erastin-treated cells. **d** Proportion of the three types of mitochondria under different treatment groups. **e**, **f** Dual-color live-cell imaging of IM and mtDNA in control and erastin-treated cells. **g** Frequency distribution histogram of the ratio of mtDNA area to mitochondrial area. Scale bars in **a**, **b** are 5 μm and enlarged images in **a, b** are 1 μm. Scale bars in **e**, **f** are 5 μm

To investigate these changes, we used erastin (MCE, US, HY-15763) to induce ferroptosis in COS7 cells and performed imaging under STED. In comparison with the control, the mitochondria displayed significant shrinkage into ellipsoids, along with reductions in mitochondrial length and the number of cristae (Fig. 6a, b). The distribution of mitochondrial length and the number of cristae in control and erastin-treated cells is presented in Fig. 6c. Statistical analysis revealed that the majority of mitochondria in erastin-induced ferroptosis exhibited shorter lengths and fewer cristae. All mitochondria were categorized into three classes based on their length and number of cristae. Mitochondria with lengths <2.5 μm and <6 cristae were divided into Class I, those with lengths of 2.5–5 μm or 6–12 cristae were divided into Class II, and those with lengths >5 μm or >12 cristae were divided into Class III. Representative mitochondria of these three classes in the control and erastin-treated groups are shown enlarged in Fig. 6a, b. ur analysis revealed that 55.7% of the mitochondria in the erastin-treated cells belonged to Class I, whereas the majority of mitochondria in control cells belonged to Class III (67.4%) (Fig. 6d). This indicates that erastin induces shrinking of mitochondrial morphology and a decrease in the number of cristae.

Dual-color imaging of mitochondrial cristae and mtDNA was conducted to observe mtDNA distribution and area ratio changes. A striking phenomenon was observed wherein all mtDNA converged at the center of mitochondria, disrupting the even distribution of mtDNA (Fig. 6e, f). The ratio of the mtDNA area to the mitochondrial area was calculated in both normal cells and those undergoing ferroptosis to plot the frequency distribution histogram (Fig. 6g). We found that the area ratio in normal cells was 0.291, while in cells undergoing ferroptosis, it increased to 0.363. Despite the significant morphological changes observed in the ferroptosis group, the statistical analysis indicated no significant change in the area distribution of mtDNA between the control and erastin-treated groups (Fig. S18). It is hypothesized that the increase in the area ratio may be attributed to mitochondrial shrinkage during ferroptosis.

These intriguing findings shed light on the changes that occur within mitochondria during ferroptosis, providing valuable insights into the relationship between mitochondrial structure and mtDNA distribution under this specific cell death. Further investigations into the molecular mechanisms driving these alterations could deepen our understanding of ferroptosis and its implications for cellular physiology and pathology.

## Discussion

In this study, we successfully developed a lipid membrane fluorogenic probe, HBmito Crimson, which possesses exceptional characteristics such as high photostability and brightness, specific localization to the mitochondrial IM, and lipid membrane fluorogenicity. Exploiting these outstanding properties, low-power STED imaging was conducted, achieving time-lapse imaging of over 500 frames within a total period of 52 min, with high spatial resolution of 40 nm. Compared to our previous work^19^, a significant breakthrough has been achieved both in terms of imaging frame quantity and image quality because of the excellent performance of HBmito Crimson. Previously, imaging capability was limited to a maximum of 200 frames. Here, this work has not only expanded the capacity substantially, but it has also allowed us to discern a multitude of crucial biological phenomena.

By using HBmito Crimson and SYBR Gold, we performed time-lapse dual-color STED/confocal super-resolution imaging of the IM and mtDNA with excellent resolution. Our investigations revealed that mtDNA exhibits an even distribution within mitochondrial networks, with a preference for habitation at the tips and branch points. This distribution pattern of mtDNA is closely linked to the unique architecture and dynamics of mitochondria. The disparity in mtDNA distribution between small individual mitochondria and interconnected mitochondrial networks was explored. Our findings suggest that mitochondria of different sizes may serve distinct functions.

Mitochondria can adapt to various external changes, both as isolated entities and in solidarity with each other in vast sprawling networks^44^. uring our observations of mitochondrial dynamics, we noticed the formation of mitochondrial networks through fusion events and generation of new branches, and first visualized the cristae dynamics and the mtDNA changes in distribution when mitochondria extend the branch. Interestingly, we found that fusion events tend to occur near mtDNA, likely experiencing less pressure during cristae remodeling. This close association likely also allows for the efficient transmission of mtDNA between different mitochondria during fusion. Additionally, our observations of mitochondrial fission revealed spatial links between mtDNA replication and fission sites, enabling proper genetic material segregation and even distribution throughout the mitochondrial network.

MtDNA plays an important role in maintaining cristae structure^48^. Conversely, maintenance of mtDNA distribution also requires normal cristae dynamics. In cases of apoptosis and ferroptosis, cristae structure is disrupted, leading to mtDNA distribution disorder. We observed the dynamic processes of IM herniation and mtDNA leakage during late apoptosis, as well as the shrinkage and fragmentation of mitochondria along with a convergence of mtDNA in ferroptosis. We propose the area ratio of mtDNA and mitochondrial as a potential marker for mitochondrial function and status. In healthy cells, >66% of the mitochondria are classified as Class III, which indicates mitochondria with longer lengths and a higher number of cristae. However, in the context of ferroptosis, this percentage dramatically decreases to <18%. This shift in distribution suggests a significant reduction in mitochondrial length and cristae number during ferroptosis, reflecting the pronounced morphological changes and mitochondrial damage associated with this mode of cell death.

Understanding the relationship between mtDNA, cristae structure, and mitochondrial function is of significant importance for unraveling the mechanisms behind various cellular processes, including cell death, metabolism, and responses to stress and disease. The direct association between mtDNA and the respiratory chain indicates that even mtDNA distribution is essential for normal respiratory activity in the cell. Conversely, uneven mtDNA distribution signifies respiratory dysfunction or a change in the cell’s physiological state^8^. The dynamic layouts between the IM and mtDNA respond to the mitochondrial function, adhering to the principle of “form follows function”^6^. Future research in this area will likely shed further light on the intricate interplay between mtDNA and cristae dynamics and their impact on cellular health and function. We plan to develop new mtDNA labeling methods that can be adapted to STED time-lapse imaging in future studies. Additionally, we intend to explore the dynamic behavior of mitochondrial proteins involved in the interaction between the IM and mtDNA, utilizing dual-color STED imaging to elucidate the underlying mechanisms^45,54^.

Overall, our findings shed light on the intricate relationship between mitochondrial membrane dynamics and mtDNA distribution. Understanding these mechanisms will contribute to unraveling the functions of mitochondria in cellular physiology, human diseases and aging^55–57^. The combination of advanced imaging techniques, such as dual-color imaging and STED nanoscopy with HBmito Crimson, shows substantial potential as diagnostic modality. The meticulous imaging provided by STED stands to facilitate early identification of pathologies underscored by mitochondrial dysfunction or mtDNA misplacement. With mtDNA being entirely maternally inherited, its association with mitochondrial genetic disorders, such as Leber hereditary optic neuropathy (LHON)^58^, has been established. Yet, prenatal diagnosis of these mtDNA diseases present significant hurdles. Application of super-resolution imaging for the analysis of amniotic fluid-derived cells could assess fetal health, leveraging mitochondrial morphology, mtDNA distribution, among other indicators, as biomarkers. This approach surely has the potential to open up new avenues in the realm of early detection of diseases and prenatal care^59,60^.

## Materials and methods

### Fluorescent probe design

Synthesis procedures for compounds and design strategies are shown in Supplementary Scheme S1. Chemical structure identification (^1^H, ^13^C NMR, and mass spectra) of compound **3** and **HBmito Crimson** are shown in Supplementary Information.

### STED super-resolution for live cells

Cells were seeded on coverslips or glass-bottomed dishes and cultured to a suitable density (24 h) at 37 °C in a 5% CO_2_ atmosphere with 95% humidity. Cells were incubated with HBmito Crimson for 10 min under the same conditions, after which images were obtained using a confocal laser-scanning microscope. STED imaging was performed using the Abberior Facility Line (Abberior Instruments GmbH, Germany) with a 637-nm laser for excitation and a 775-nm pulsed laser for STED depletion. A 60× oil-immersion objective (N.A. 1.42, Olympus, Japan) was employed in imaging experiments. All STED results presented in the paper have been processed by Huygens deconvolution software. The deconvolution parameter is set to improve the resolution while maintaining the real structure. The acquisition parameters of the microscope are shown in Table S2.

#### STED two-color imaging

The cells were incubated in DMEM solution containing SYBR Gold (1000×) for 20 min. Following the incubation, the cells were cleaned three times using a PBS solution. Subsequently, the cells were incubated with DMEM solution containing 500 nM HBmito Crimson for 15 min. After the incubation, the cells were ready for wash-free STED imaging.

### Spinning-disk confocal microscopy

Spinning disk confocal data were acquired under a Yokogawa spinning disk equipped with a Live SR super-resolution module (Gataca systems, France), enabling a 2× resolution improvement at very high imaging speeds. A 100× oil-immersion objective (N.A. 1.4, Nikon, Japan) was used in live cell imaging.

### Segmentation of mitochondrial cristae and statistical analyses

The areas of mitochondrial cristae and mtDNA were calculated using ImageJ plugins. We applied the ImageJ plugin Trainable Weka Segmentation to train the cristae classifier and obtain probability images^61^. The threshold was adjusted until cristae were accurately distinguished from the background, and binary images were then obtained (Fig. S19). For binary images, mitochondrial and mtDNA areas were calculated using the analysis function in ImageJ.

#### Apoptosis induction

COS7 cells were treated with 10 μM ABT-737 and 2 μM S63845 for 30 min in the HBSS system, incubated at 37 °C in a 5% CO_2_ atmosphere with 95% humidity, washed three times with HBSS, and then subjected to staining according to the previously established two-color imaging protocol.

#### Ferroptosis induction

The cells were labeled with HBmito Crimson at a concentration of 500 nM and SYBR Gold at a dilution of 1000×. Subsequently, the cells were washed three times with PBS and then incubated in DMEM containing Erastin (2 μM) for a duration of 10 min.

#### STED saturation power measurement

Dilute the HBmito Crimson dye concentration to 5 μM with DMSO, pipette ~1 mL, and add it to the confocal cuvette. Set the SLM (775 nm) Mode to “none” in the background interface of the commercial STED system, transforming the depletion beam of the donut distribution into a gaussian distribution. Adjust the excitation intensity to 3% of the total laser intensity (calibrated based on the dye brightness), and gradually increase the depletion laser intensity to image the dye molecules in the solution state. Next, set the excitation light intensity to 0% and gradually increase the depletion laser intensity to image the dye molecules in the solution state for a second time. The saturated intensity of the dye can be determined by plotting all the data obtained by subtracting the average fluorescence intensity of the image in the second imaging session from that of the first under the same depletion laser power, normalizing, and fitting it to a curve. Identify the point at which the fluorescence intensity drops to 50% (indicating a depletion efficiency of 50%); the corresponding depletion power is the saturation power of the dye.

## Supplementary information

Supplementary Information for: Visualization of cristae and mtDNA interactions via STED nanoscopy using a low saturation power probe

Movie S1

Movie S2

Movie S3

Movie S4

Movie S5

Movie S6

Movie S7

Movie S8

Movie S9

Movie S10

## Data Availability

The data underlying the results presented in this paper is available from the corresponding author upon reasonable request.

## References

[1] Spinelli, J. B. & Haigis, M. C. The multifaceted contributions of mitochondria to cellular metabolism. *Nat. Cell Biol.* **20**, 745–754 (2018). 29950572 10.1038/s41556-018-0124-1 PMC6541229

[2] Tan, J. X. & Finkel, T. Mitochondria as intracellular signaling platforms in health and disease. *J. Cell Biol.* **219**, e202002179 (2020). 32320464 10.1083/jcb.202002179 PMC7199861

[3] Rizzuto, R. et al. Mitochondria as sensors and regulators of calcium signalling. *Nat. Rev. Mol. Cell Biol.* **13**, 566–578 (2012). 22850819 10.1038/nrm3412

[4] Lill, R. et al. The role of mitochondria in cellular iron-sulfur protein biogenesis and iron metabolism. *Biochim. Biophys. Acta (BBA) Mol. Cell Res.* **1823**, 1491–1508 (2012). 10.1016/j.bbamcr.2012.05.009 22609301

[5] Friedman, J. R. & Nunnari, J. Mitochondrial form and function. *Nature* **505**, 335–343 (2014). 24429632 10.1038/nature12985 PMC4075653

[6] Cogliati, S., Enriquez, J. A. & Scorrano, L. Mitochondrial cristae: where beauty meets functionality. *Trends Biochem. Sci.* **41**, 261–273 (2016). 26857402 10.1016/j.tibs.2016.01.001

[7] Baker, N., Patel, J. & Khacho, M. Linking mitochondrial dynamics, cristae remodeling and supercomplex formation: how mitochondrial structure can regulate bioenergetics. *Mitochondrion* **49**, 259–268 (2019). 31207408 10.1016/j.mito.2019.06.003

[8] Chapman, J., Ng, Y. S. & Nicholls, T. J. The maintenance of mitochondrial DNA integrity and dynamics by mitochondrial membranes. *Life* **10**, 164 (2020). 32858900 10.3390/life10090164 PMC7555930

[9] Richter-Dennerlein, R. et al. Mitochondrial protein synthesis adapts to influx of nuclear-encoded protein. *Cell* **167**, 471–483.e10 (2016). 27693358 10.1016/j.cell.2016.09.003 PMC5055049

[10] Kopek, B. G. et al. Correlative 3D superresolution fluorescence and electron microscopy reveal the relationship of mitochondrial nucleoids to membranes. *Proc. Natl Acad. Sci. USA* **109**, 6136–6141 (2012). 22474357 10.1073/pnas.1121558109 PMC3341004

[11] Gilkerson, R. W. et al. Mitochondrial nucleoids maintain genetic autonomy but allow for functional complementation. *J. Cell Biol.* **181**, 1117–1128 (2008). 18573913 10.1083/jcb.200712101 PMC2442202

[12] Ban-Ishihara, R. et al. Dynamics of nucleoid structure regulated by mitochondrial fission contributes to cristae reformation and release of cytochrome c. *Proc. Natl Acad. Sci. USA* **110**, 11863–11868 (2013). 23821750 10.1073/pnas.1301951110 PMC3718159

[13] Liu, T. Y. et al. Multi-color live-cell STED nanoscopy of mitochondria with a gentle inner membrane stain. *Proc. Natl Acad. Sci. USA* **119**, e2215799119 (2022). 36534799 10.1073/pnas.2215799119 PMC9907107

[14] Stephan, T. et al. Live-cell STED nanoscopy of mitochondrial cristae. *Sci. Rep.* **9**, 12419 (2019). 31455826 10.1038/s41598-019-48838-2 PMC6712041

[15] Wang, C. G. et al. A photostable fluorescent marker for the superresolution live imaging of the dynamic structure of the mitochondrial cristae. *Proc. Natl Acad. Sci. USA* **116**, 15817–15822 (2019). 31337683 10.1073/pnas.1905924116 PMC6689947

[16] Jakobs, S. et al. Light microscopy of mitochondria at the nanoscale. *Annu. Rev. Biophys.* **49**, 289–308 (2020). 32092283 10.1146/annurev-biophys-121219-081550 PMC7610798

[17] Shim, S. H. et al. Super-resolution fluorescence imaging of organelles in live cells with photoswitchable membrane probes. *Proc. Natl Acad. Sci. USA* **109**, 13978–13983 (2012). 22891300 10.1073/pnas.1201882109 PMC3435176

[18] Huang, X. S. et al. Fast, long-term, super-resolution imaging with Hessian structured illumination microscopy. *Nat. Biotechnol.* **36**, 451–459 (2018). 29644998 10.1038/nbt.4115

[19] Yang, X. S. et al. Mitochondrial dynamics quantitatively revealed by STED nanoscopy with an enhanced squaraine variant probe. *Nat. Commun.* **11**, 3699 (2020). 32709877 10.1038/s41467-020-17546-1 PMC7382495

[20] Qiao, C. et al. Evaluation and development of deep neural networks for image super-resolution in optical microscopy. *Nat. Methods* **18**, 194–202 (2021). 33479522 10.1038/s41592-020-01048-5

[21] Chen, X. et al. Superresolution structured illumination microscopy reconstruction algorithms: a review. *Light Sci. Appl.* **12**, 172 (2023). 37433801 10.1038/s41377-023-01204-4 PMC10336069

[22] Cao, R. J. et al. Open-3DSIM: an open-source three-dimensional structured illumination microscopy reconstruction platform. *Nat. Methods* **20**, 1183–1186 (2023). 37474809 10.1038/s41592-023-01958-0 PMC10406603

[23] Qian, J. M. et al. Structured illumination microscopy based on principal component analysis. *eLight* **3**, 4 (2023). 10.1364/OL.480330 36563399

[24] Zhao, K. et al. Two-photon MINFLUX with doubled localization precision. *eLight* **2**, 5 (2022).

[25] Song, Y. F. et al. Improving brightness and stability of Si-rhodamine for super-resolution imaging of mitochondria in living cells. *Anal. Chem.* **92**, 12137–12144 (2020). 32844652 10.1021/acs.analchem.9b04926

[26] Yang, X. S. et al. Mirror-enhanced super-resolution microscopy. *Light Sci. Appl.* **5**, e16134 (2016). 27398242 10.1038/lsa.2016.134 PMC4936537

[27] Xu, X. Z. & Xi, P. Rare nanoparticles shine colors with low-power STED. *Light Sci. Appl.* **11**, 171 (2022). 35668074 10.1038/s41377-022-00863-z PMC9170770

[28] Johnson, L. V., Walsh, M. L. & Chen, L. B. Localization of mitochondria in living cells with rhodamine 123. *Proc. Natl Acad. Sci. USA* **77**, 990–994 (1980). 6965798 10.1073/pnas.77.2.990 PMC348409

[29] Siewert, B. et al. Turning on the red phosphorescence of a [Ru (tpy)(bpy)(Cl)] Cl complex by amide substitution: self-aggregation, toxicity, and cellular localization of an emissive ruthenium-based amphiphile. *Chem. Commun.* **53**, 11126–11129 (2017). 10.1039/c7cc02989f 28682371

[30] Zhang, H. K. et al. Aggregate science: from structures to properties. *Adv. Mater.* **32**, 2001457 (2020). 10.1002/adma.202001457 32734656

[31] Morgan, M. T., McCallum, A. M. & Fahrni, C. J. Rational design of a water-soluble, lipid-compatible fluorescent probe for Cu(I) with sub-part-per-trillion sensitivity. *Chem. Sci.* **7**, 1468–1473 (2016). 28042469 10.1039/c5sc03643g PMC5201193

[32] Rajakumar, D. V. & Rao, M. N. A. Dehydrozingerone and isoeugenol as inhibitors of lipid peroxidation and as free radical scavengers. *Biochem. Pharmacol.* **46**, 2067–2072 (1993). 8267655 10.1016/0006-2952(93)90649-h

[33] Yang, Z. T. et al. Cyclooctatetraene-conjugated cyanine mitochondrial probes minimize phototoxicity in fluorescence and nanoscopic imaging. *Chem. Sci.* **11**, 8506–8516 (2020). 34094186 10.1039/d0sc02837a PMC8161535

[34] Rittweger, E. et al. Fluorescence depletion mechanisms in super-resolving STED microscopy. *Chem. Phys. Lett.* **442**, 483–487 (2007).

[35] Vicidomini, G. et al. STED with wavelengths closer to the emission maximum. *Opt. Express* **20**, 5225–5236 (2012). 22418329 10.1364/OE.20.005225

[36] Koho, S. et al. Fourier ring correlation simplifies image restoration in fluorescence microscopy. *Nat. Commun.* **10**, 3103 (2019). 31308370 10.1038/s41467-019-11024-z PMC6629685

[37] Zheng, S. et al. Long-term super-resolution inner mitochondrial membrane imaging with a lipid probe. *Nat. Chem. Biol.* **20**, 83–92 (2024). 37857992 10.1038/s41589-023-01450-y PMC10746544

[38] Orrenius, S., Zhivotovsky, B. & Nicotera, P. Regulation of cell death: the calcium-apoptosis link. *Nat. Rev. Mol. Cell Biol.* **4**, 552–565 (2003). 12838338 10.1038/nrm1150

[39] Kilian, N. et al. Assessing photodamage in live-cell STED microscopy. *Nat. Methods* **15**, 755–756 (2018). 30275592 10.1038/s41592-018-0145-5 PMC6915835

[40] Osman, C. et al. Integrity of the yeast mitochondrial genome, but not its distribution and inheritance, relies on mitochondrial fission and fusion. *Proc. Natl Acad. Sci. USA* **112**, E947–E9956 (2015). 25730886 10.1073/pnas.1501737112 PMC4352819

[41] Lewis, S. C., Uchiyama, L. F. & Nunnari, J. ER-mitochondria contacts couple mtDNA synthesis with mitochondrial division in human cells. *Science* **353**, eaaf5549 (2016). 10.1126/science.aaf5549 PMC5554545 27418514

[42] Jajoo, R. et al. Accurate concentration control of mitochondria and nucleoids. *Science* **351**, 169–172 (2016). 26744405 10.1126/science.aaa8714 PMC4823142

[43] Wang, C. et al. Dynamic tubulation of mitochondria drives mitochondrial network formation. *Cell Res.* **25**, 1108–1120 (2015). 26206315 10.1038/cr.2015.89 PMC4650629

[44] Chan, D. C. Fusion and fission: interlinked processes critical for mitochondrial health. *Annu. Rev. Genet.* **46**, 265–287 (2012). 22934639 10.1146/annurev-genet-110410-132529

[45] Qin, J. S. et al. ER-mitochondria contacts promote mtDNA nucleoids active transportation via mitochondrial dynamic tubulation. *Nat. Commun.* **11**, 4471 (2020). 32901010 10.1038/s41467-020-18202-4 PMC7478960

[46] Kondadi, A. K. et al. Cristae undergo continuous cycles of membrane remodelling in a MICOS-dependent manner. *EMBO Rep.* **21**, e49776 (2020). 32067344 10.15252/embr.201949776 PMC7054676

[47] Murley, A. et al. ER-associated mitochondrial division links the distribution of mitochondria and mitochondrial DNA in yeast. *eLife* **2**, e00422 (2013). 23682313 10.7554/eLife.00422 PMC3654481

[48] Blum, T. B. et al. Dimers of mitochondrial ATP synthase induce membrane curvature and self-assemble into rows. *Proc. Natl Acad. Sci. USA* **116**, 4250–4255 (2019). 30760595 10.1073/pnas.1816556116 PMC6410833

[49] Bock, F. J. & Tait, S. W. G. Mitochondria as multifaceted regulators of cell death. *Nat. Rev. Mol. Cell Biol.* **21**, 85–100 (2020). 31636403 10.1038/s41580-019-0173-8

[50] McArthur, K. et al. BAK/BAX macropores facilitate mitochondrial herniation and mtDNA efflux during apoptosis. *Science* **359**, eaao6047 (2018). 29472455 10.1126/science.aao6047

[51] Dixon, S. J. et al. Ferroptosis: an iron-dependent form of nonapoptotic cell death. *Cell* **149**, 1060–1072 (2012). 22632970 10.1016/j.cell.2012.03.042 PMC3367386

[52] Gao, M. H. et al. Role of mitochondria in ferroptosis. *Mol. Cell* **73**, 354–363.e3 (2019). 30581146 10.1016/j.molcel.2018.10.042 PMC6338496

[53] DeHart, D. N. et al. Opening of voltage dependent anion channels promotes reactive oxygen species generation, mitochondrial dysfunction and cell death in cancer cells. *Biochem. Pharmacol.* **148**, 155–162 (2018). 29289511 10.1016/j.bcp.2017.12.022 PMC5909406

[54] Stoldt, S. et al. Mic60 exhibits a coordinated clustered distribution along and across yeast and mammalian mitochondria. *Proc. Natl Acad. Sci. USA* **116**, 9853–9858 (2019). 31028145 10.1073/pnas.1820364116 PMC6525524

[55] Nunnari, J. & Suomalainen, A. Mitochondria: in sickness and in health. *Cell* **148**, 1145–1159 (2012). 22424226 10.1016/j.cell.2012.02.035 PMC5381524

[56] Trifunovic, A. et al. Premature ageing in mice expressing defective mitochondrial DNA polymerase. *Nature* **429**, 417–423 (2004). 15164064 10.1038/nature02517

[57] Kujoth, G. C. et al. Mitochondrial DNA mutations, oxidative stress, and apoptosis in mammalian aging. *Science* **309**, 481–484 (2005). 16020738 10.1126/science.1112125

[58] Zuccarelli, M. et al. Treatment of Leber’s hereditary optic neuropathy: an overview of recent developments. *Eur. J. Ophthalmol.* **30**, 1220–1227 (2020). 32552047 10.1177/1120672120936592

[59] Dong, S. L. et al. Early cancer detection by serum biomolecular fingerprinting spectroscopy with machine learning. *eLight* **3**, 17 (2023).

[60] Shi, L. Y., Li, Y. J. & Li, Z. Early cancer detection by SERS spectroscopy and machine learning. *Light Sci. Appl.* **12**, 234 (2023). 37714845 10.1038/s41377-023-01271-7 PMC10504315

[61] Segawa, M. et al. Quantification of cristae architecture reveals time-dependent characteristics of individual mitochondria. *Life Sci. Alliance* **3**, e201900620 (2020). 32499316 10.26508/lsa.201900620 PMC7283135

