# Author Correction: Visualization of cristae and mtDNA interactions via STED nanoscopy using a low saturation power probe

**DOI:** 10.1038/s41377-024-01584-1

**Published:** 2024-09-05

**Authors:** Wei Ren, Xichuan Ge, Meiqi Li, Jing Sun, Shiyi Li, Shu Gao, Chunyan Shan, Baoxiang Gao, Peng Xi

**Affiliations:** 1 Department of Biomedical Engineering, National Biomedical Imaging Center, College of Future Technology, Peking University, Beijing, 100871 China; 2 Key Laboratory of Analytical Science and Technology of Hebei Province, College of Chemistry and Material Science, Hebei University, Baoding, 071002 China; 3 School of Life Sciences, Peking University, Beijing, 100871 China; 4 National Center for Protein Sciences, Peking University, Beijing, 100871 China

**Keywords:** Super-resolution microscopy, Biophotonics

Correction to: *Light: Science & Applications*

10.1038/s41377-024-01463-9 published online 24 May 2024

After publication of this article^1^, it was brought to our attention that the unit of NaCl in the legend “NaCl (150 μM) + Liposome” in Fig. 1f was incorrect and should be “NaCl (150 mM) + Liposome”. The original publication has been corrected. The correct Fig. 1 is shown below:
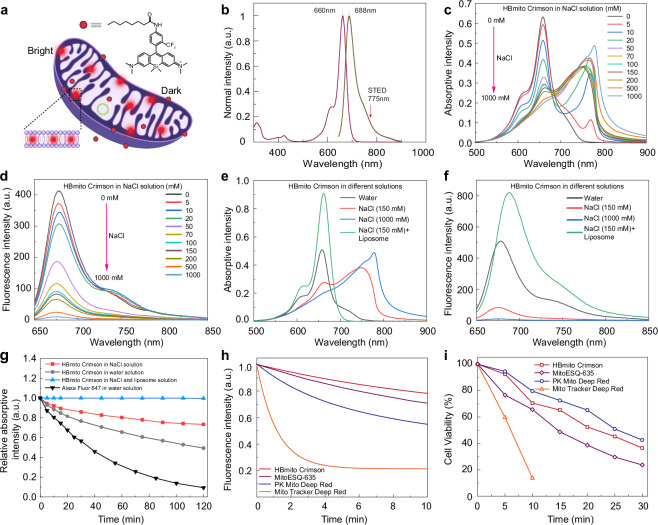


**Fig. 1: A lipid membrane light-up, highly photostable, lowly phototoxicity mitochondrial inner membrane probe. a** Chemical structure of HBmito Crimson used for the specific labeling of the mitochondrial inner membrane. **b** The absorption and emission spectra of HBmito Crimson, which can be depleted using a 775-nm laser. **c**, **d** Absorption and fluorescence spectra of HBmito Crimson solution upon addition of different concentrations of NaCl (0–1000 mM). **e**, **f** Absorption and fluorescence spectra of HBmito Crimson solution in NaCl solution in the presence or absence of liposome. **g** The photostability of HBmito Crimson in different solutions and commercial Alexa Fluor 647 in water solution. **h** Photobleaching curve of HBmito Crimson, PK Mito Deep Red and Mito Tracker Deep Red in polymethyl methacrylate (PMMA). **i** Viability of HBmito Crimson, PK Mito Deep Red and Mito Tracker Deep Red-stained COS7 cells after Red light illumination (637 nm, 1.6 W/cm^2^).

## References

[1] Ren, W. et al. Visualization of cristae and mtDNA interactions via STED nanoscopy using a low saturation power probe. *Light Sci. Appl.* **13**, 116 (2024). 38782912 10.1038/s41377-024-01463-9 PMC11116397

