# Supplementary material for: Visualization of cristae and mtDNA interactions via STED nanoscopy using a low saturation power probe
Source: Light Sci Appl. 2024 May 24;13:116. doi: 10.1038/s41377-024-01463-9 (PMC11116397; doi:10.1038/s41377-024-01463-9)
Supplement: Supplementary file 1 — Supplementary Information for: Visualization of cristae and mtDNA interactions via STED nanoscopy using a low saturation power probe [file 41377_2024_1463_MOESM1_ESM.pdf]

Supplementary Information for:

Visualization of cristae and mtDNA interactions via STED nanoscopy using a low saturation power probe

Wei Ren[1,3,†], Xichuan Ge[2, †], Meiqi Li[4], Jing Sun[2], Shiyi Li[2], Shu Gao[1,3], Chunyan Shan[4,5*], Baoxiang Gao[2,*], Peng Xi[1,3,*]

[1] *Department of Biomedical Engineering, College of Future Technology, Peking University, Beijing 100871, China*

[2] *Key Laboratory of Analytical Science and Technology of Hebei Province, College of Chemistry and Material Science, Hebei University. Baoding, 071002, China.*

[3] *National Biomedical Imaging Center, Peking University, Beijing 100871, China*

[4] *School of Life Sciences, Peking University, Beijing, 100871, China*

[5] *National Center for Protein Sciences, Peking University, Beijing, 100871, China*

**Correspondence: Chunyan Shan (chunyanshan@pku.edu.cn) or Baoxiang Gao (bxgao@hbu.edu.cn) or Peng Xi (xipeng@pku.edu.cn)*

† *These authors contributed equally: Wei Ren, Xichuan Ge*

Contents

Supplementary Methods …………………………………………………….……….. 3
Supplementary Chemical Synthesis ……………………………………………………. 6
Supplementary Figures …………………………………………………..…………… 8
Supplementary Tables …………………………………………………………..…… 20
Supplementary Movies ……………………………………………………………… 23
NMR spectra of newly synthesized compounds and Mass spectra analysis …………... 24

Supplementary Methods

UV/Vis absorption and fluorescence spectroscopy measurements. Si-rhodamine probe was dissolved in DMSO to prepare a stock solution (10 mM) and used at a working concentration of 10 μM. UV/Vis absorption spectra of HBmito Crimson were measured on Specord 210 plus spectrophotometer (Analytikjena, German), and fluorescence emission spectrum was measured with Hitachi F-7000 fluorescence spectrophotometer (Hitachi, Japan). All measurements were performed in a 3-mL cuorvette with 2 mL solution. The absorption and fluorescence spectra of probe HBmito Crimson (10 μM) were measured in the following solutions. (1) Sodium chloride solution (150 μM) and Artificial lipid membrane solution (2 mM). (2) Different concentrations of sodium chloride solution (2 mM，5 mM，10 mM，20 mM，30 mM，50 mM，70 mM，100 mM，154 mM，200 mM，500 mM, and 1000 mM). Dimyristyl phosphatidyl choline (DMPC) (32.3 mg) and dimyristyl phosphatidyl glycerophosphate (DMPG) (8.21 mg) were dissolved in 20 mL mixture solution (MeOH/DCM = 1/4, v/v). After evaporating solvent and drying for 2 h under reduced pressure, the solid was dissolved in 28.9 mL sodium chloride solution (100 mM), further passed argon for 10 min to remove the dissolved oxygen in the solution, ultrasonic 5 min, passed aqueous polycarbonate membrane (200 nm) for 21 times.

Fluorescence quantum yield measurements. The fluorescent quantum yields for Probe Si-rhodamines were measured in DMSO using Alexa Fluor 647 (Φ = 0.27, in PBS) as the standard substance at an excitation wavelength of 646 nm, and the quantum yields were calculated using the following equation: $\Phi_s = \Phi_r (A_rF_s/A_sF_r)(n_s^2/n_r^2)$, where s and r denote sample and reference, respectively, A is the absorbance, F is the relative integrated fluorescence intensity, and n is the refractive index of the solvent.

Dynamic light scattering measurements. Dynamic light scattering (DLS) was determined with Winner 802 (Jnwinner, China). The diameter of aggregates of probe HBmito Crimson (10 μM) in 3 mL sodium chloride solution of different concentrations (0 mM, 20 mM, 50 mM, 100 mM, and 200 mM) was determined using dynamic light scattering methods.

Photon-stability measurements. The photon-stability of probe HBmito Crimson (10 μM) and Alexa Fluor 647 in different solutions including H_2O, NaCl solution (10 mM) and artificial lipid membrane solution (2 mM) were irradiated with 1 Watt 660 nm LED laser (1 Watt on sample). Absorption spectra were measured after irradiation, and relative

absorbance at the maximum wavelength was plotted as a function of irradiation time with a laser.

Photon-bleaching measurements. Dyes were immobilized within polymethyl methacrylate (PMMA) films prepared using a KW-4A spin coater (IMECAS, Beijing, China) and round glass coverslips (diameter = 0.17 mm) as substrates. 100 μL 5% PMMA in Tetrahydrofuran containing 2 μM dyes were applied to coverslip followed by spin coating(1200 rpm 10 sec; 6300 rpm 30 sec). These polymer films were then irradiated using a Spinning-disk confocal microscopy (Nikon, Japanese) equipped with a 63× Oil/1.4 N.A. objective. Three groups of time-lapse images (500 frames, 108 μm×108 μm, 5 fps, 100% laser power) were acquired for each dye, and quantification of the fluorescence intensity was achieved via Analyze >> Tools >> ROI manager in the Fiji software from three parallel experiments.

Phototoxicity assay. Live COS7 cells were labeled with HBmito Crimson at a concentration of 500 nM. The cells were then exposed to light at an intensity of 1.4 W/cm^2 for a specific duration. Then the cells recovered in the incubator for 30 min. Following the light exposure, the cells were treated with Calcein AM. Subsequently, the cells that lacked a green signal were identified as dead cells and quantified. The experimental protocol involved analyzing hundreds of cells using a 20× objective lens.

Cell culture. COS7 cells were cultured at a suitable density (moderate) in DMEM supplemented with penicillin (100 units/mL), 10% (v/v) FBS (WelGene), and streptomycin (100 mg/mL) at 37°C in a 5% CO_2 atmosphere with 95% humidity. Cells were seeded on confocal dishes 24 h prior to the experiments.

Cytoplasmic calcium level measurements. Cells were first incubated in HBSS solution containing 2 μM Fluo4 for 20 minutes, followed by a subsequent 20-minute incubation in new HBSS solution. Subsequently, 500 nM HBmito Crimson was added and imaged. Ionomycin (IO) was employed as a positive control for Ca^{2+} release experiments. IO acts as a selective Ca^{2+} ionophore, making the cellular membranes highly Ca^{2+} permeable, inducing a rapid surge in cytoplasmic Ca^{2+} concentration. IO diluted to 20 μM in imaging buffer was added to COS7 cells. The calcium response of the cells was monitored both before and after the administration of IO.

The image results were obtained on a STEDYCON microscope equipped with a 100×/1.42N.A. oil lens. The duration of a frame is about 12s, and the total time is about 12min. The Fluo4 signal was imaged in the first frame of each imaging cycle with the

488nm laser. The Confocal irradiation experiment was filmed in confocal mode with the same other parameters as the STED irradiation experiment.

Supplementary Chemical Synthesis

Materials and characterization. All chemicals used for synthesis were purchased from commercial suppliers and applied directly in the experiment without further purification. Solvents were either employed as purchased or dried according to procedures described in the literature. The progress of the reaction was monitored by TLC on pre-coated silica plates (GF-254, 250 μm in thickness), and spots were visualized by UV light. Qingdao ocean silica gel (100-200 mesh) was used for general column chromatography purification. ^{1}H NMR and ^{13}C NMR spectra were recorded on Q. One AS 400 or Bruker 600 spectrometer with $CDCl_3$, DMSO-d_6 or CD_3OD as solvent. Chemical shifts are reported in parts per million relative to internal standard tetramethylsilane ($Si(CH_3)_4$ = 0.00 ppm). High-resolution mass spectra (HRMS) were obtained on a XEVO-G2QTOF (ESI) (Waters, USA) or TSQ Quantum Ultra (ESI) (Thermos, Germany).

Synthesis

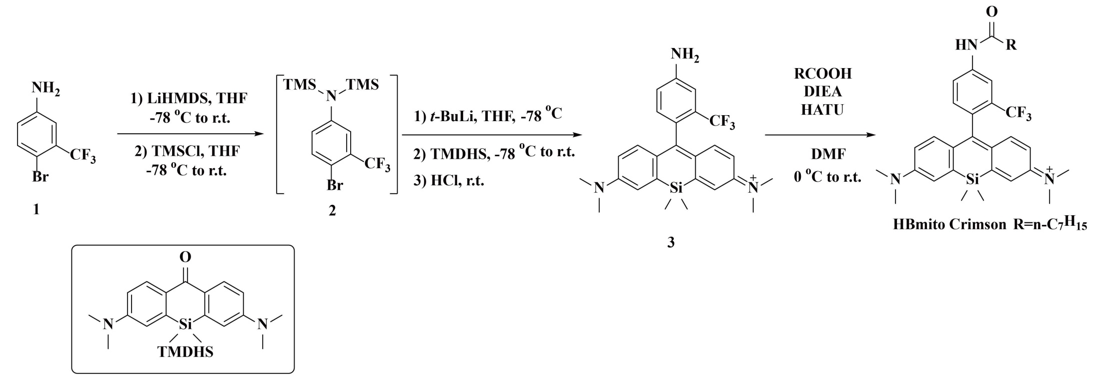

Scheme S1 Synthetic scheme for Si-rhodamines.

Synthesis of compound 2. In a nitrogen-flushed flask fitted with a double port reaction bottle, compound **1** (360 mg, 1.5 mmol) was dissolved in anhydrous THF (10 mL) and the solution was cooled to -78 °C. 1.3 M Lithium bis(trimethylsilyl)amide (LiHMDS) 2.5 mL, 3.3 mmol) was slowly added dropwise via a syringe to the above solution in an N_2 atmosphere. After that, the reaction solution was stirred for 20 minutes at -78 °C, then warmed to room temperature, and further stirred for 5 min. After further cooling to -78 °C, dimethyldichlorosilane (TMSCl) (359 mg, 3.3 mmol) dissolved in anhydrous THF was slowly added into the system, then the solution was warmed to room temperature and stirred for 16 h. The solvent was evaporated at reduced pressure to obtain intermediate **2** without separation, which was used directly for the next step.

Synthesis of compound 3. In a nitrogen-flushed flask fitted with a double port reaction bottle, intermediate **2** was dissolved in anhydrous THF (10 mL) and the solution was cooled

to -78 °C. 1.3 M *t*-BuLi (1.1 mL, 1.5 mmol) was slowly added dropwise via a syringe to the above solution in an N_2 atmosphere. After that, the reaction solution was stirred for 30 minutes at -78 °C. TMDHS (50 mg, 0.15 mmol) dissolved in anhydrous THF was slowly added into the system, then the solution was warmed to room temperature and stirred for 2 h, quenched with 2 M HCl. The aqueous solution was extracted with dichloromethane, and the combined organic phase was washed with water and brine, dried over Na_2SO_4, filtered and evaporated. The resultant residue was quickly purified by silica gel chromatography (MeOH/DCM = 1/50, v/v), yielded 74%, 53 mg of pure product as blue solid. ^{1}H NMR (400 MHz, $CDCl_3$) δ 7.14 (d, J = 10.2 Hz, 2H), 7.09 (d, J = 8.7 Hz, 4H), 6.84 (d, J = 8.2 Hz, 1H), 6.56 (dd, J = 9.6, 2.3 Hz, 2H), 3.34 (s, 12H), 0.59 (s, 3H), 0.46 (s, 3H). ^{13}C NMR (101 MHz, $CDCl_3$) δ 168.94, 153.89, 148.80, 148.10, 142.40, 131.47, 128.81, 125.14, 123.77, 122.41, 120.26, 117.43, 113.49, 111.83, 77.48, 77.16, 76.84, 40.99, 29.61, -0.27, -1.93. MALDI-TOF MS m/z Calculated 468.2077 for $C_{26}H_{29}F_3N_3Si^+$, found 468.1682 $[M]^+$.

Synthesis of probe HBmito Crimson. In a flame-dried flask flushed with nitrogen, n-octylic acid (17.3 mg, 0.12 mmol) was dissolved in anhydrous DMF (10 mL). HATU (55 mg, 0.144 mmol) was added at ice bath. After stirred at room temperature for 0.5 h, compound **3** (28 mg, 0.06 mmol) and DIPEA (31 mg, 0.24 mmol) were added one by one at ice bath. After further stirred for 3 h, the reaction solution was extracted with dichloromethane. The organic layer was dried over Na_2SO_4 and evaporated. The resultant residue was quickly purified by silica gel chromatography (MeOH/DCM = 1/100, v/v), yielded 84%, 30 mg of pure product as blue solid. ^{1}H NMR (400 MHz, DMSO-d_6) δ 10.47 (s, 1H), 8.30 (s, 1H), 7.98 (d, J = 8.3 Hz, 1H), 7.42 (s, 2H), 7.35 (d, J = 8.2 Hz, 1H), 6.84 (q, J = 9.7 Hz, 4H), 3.31 (s, 12H), 2.40 (t, J = 7.0 Hz, 2H), 1.64 (s, 2H), 1.30 (d, J = 14.0 Hz, 8H), 0.90 - 0.83 (m, 4H), 0.64 (s, 3H), 0.51 (s, 3H). ^{13}C NMR (101 MHz, $CDCl_3$) δ 173.88, 167.56, 154.45, 148.78, 142.68, 140.42, 131.65, 131.22, 130.35, 128.77, 122.99, 120.90, 118.22, 114.12, 77.80, 77.48, 77.16, 41.29, 37.72, 32.37, 32.18, 30.15, 27.66, 25.95, 23.10, 14.58, 0.04, -1.73. MALDI-TOF MS m/z Calculated 594.3122 for $C_{34}H_{43}F_3N_3OSi^+$, found 594.3130 $[M]^+$.

Supplementary Figures

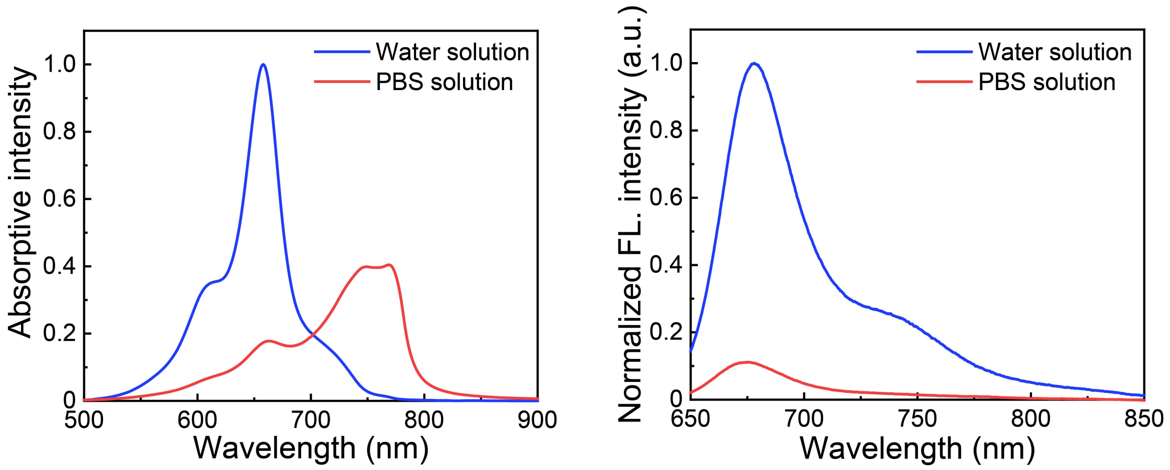

Fig. S1 The absorption and emission spectra of HBmito Crimson in H_2O and PBS solutions.

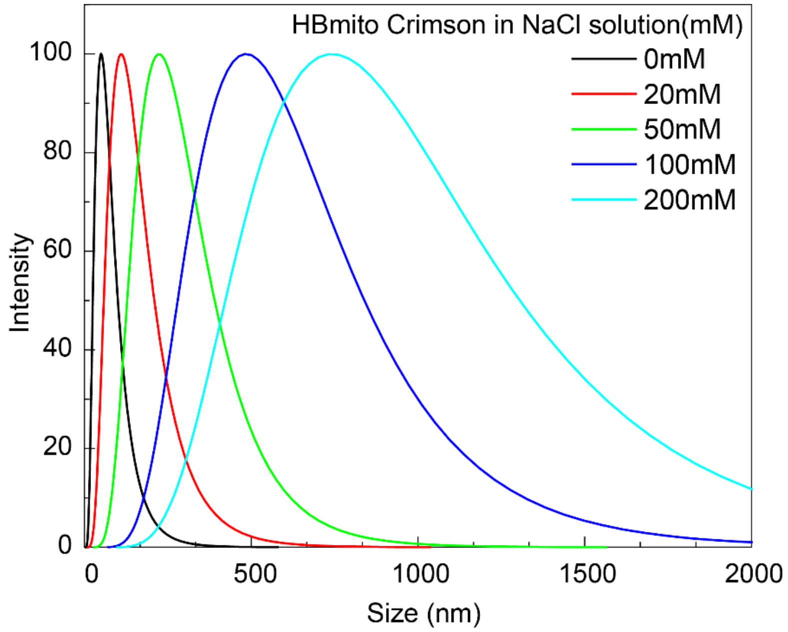

Fig. S2 The particle size distribution of HBmito Crimson in different concentrations of NaCl solution.

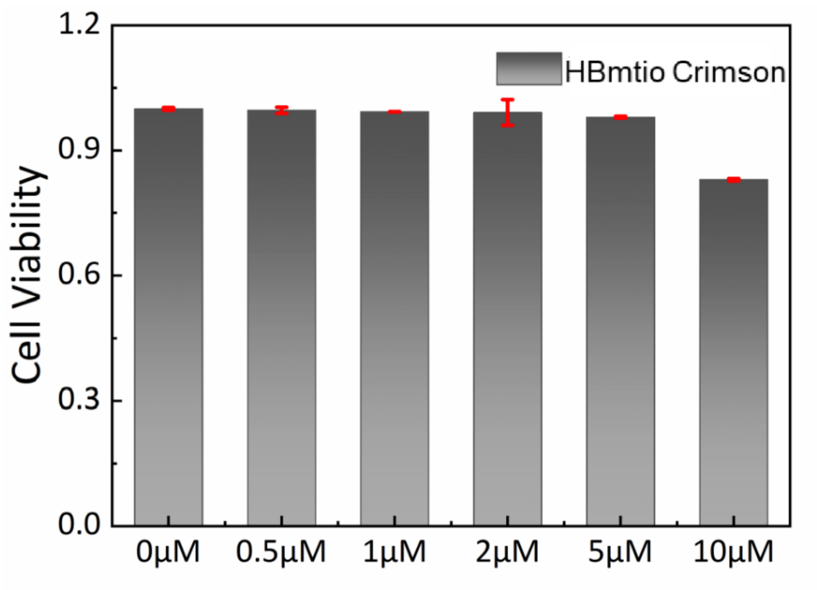

Fig. S3 The cytotoxicity of probe HBmito Crimson using a standard MTT assay.

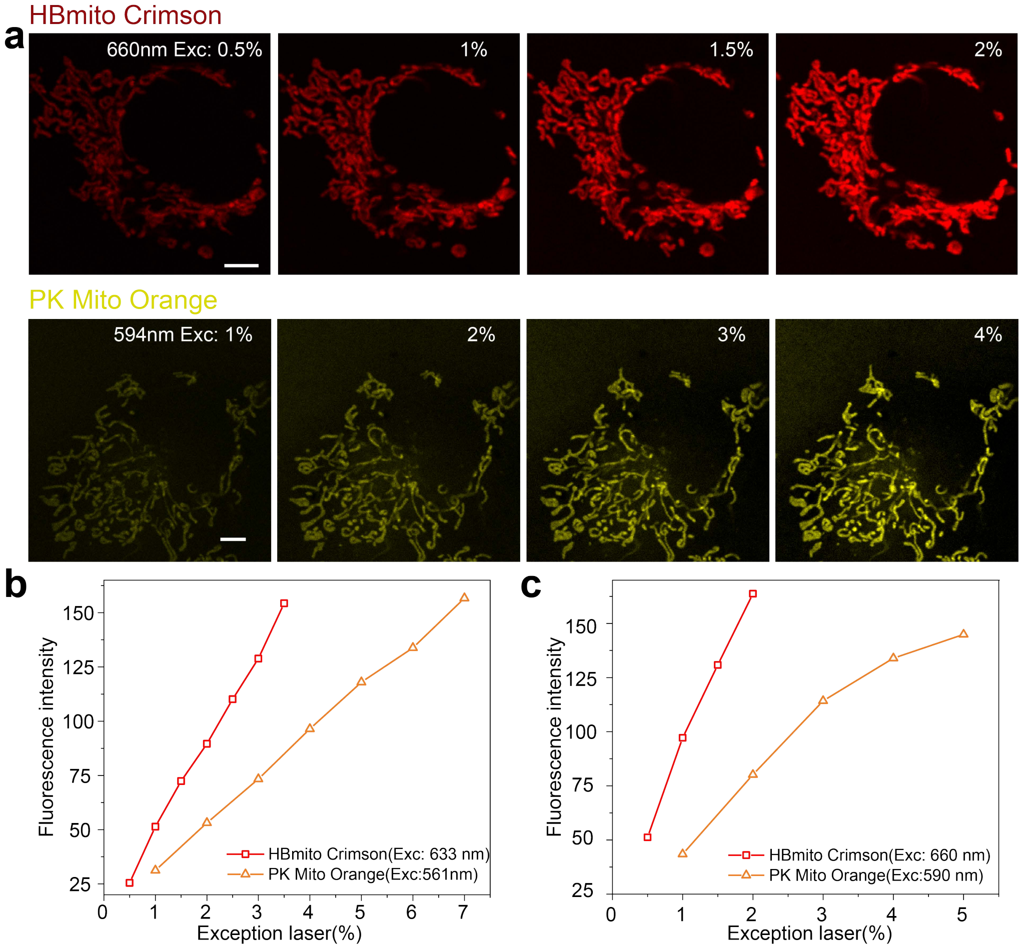

Fig. S4 a Brightness comparison of HBmito Crimson and PK Mito Orange in living cell at different excitation powers (shown in the same range). **b** Power comparison at the

excitation bands most commonly set of commercial microscopes, by averaging the signals in selected labeled cells. **c** Power comparison at the respective optimal excitation bands, by averaging the signals in selected labeled cells. Scales are 5 μm.

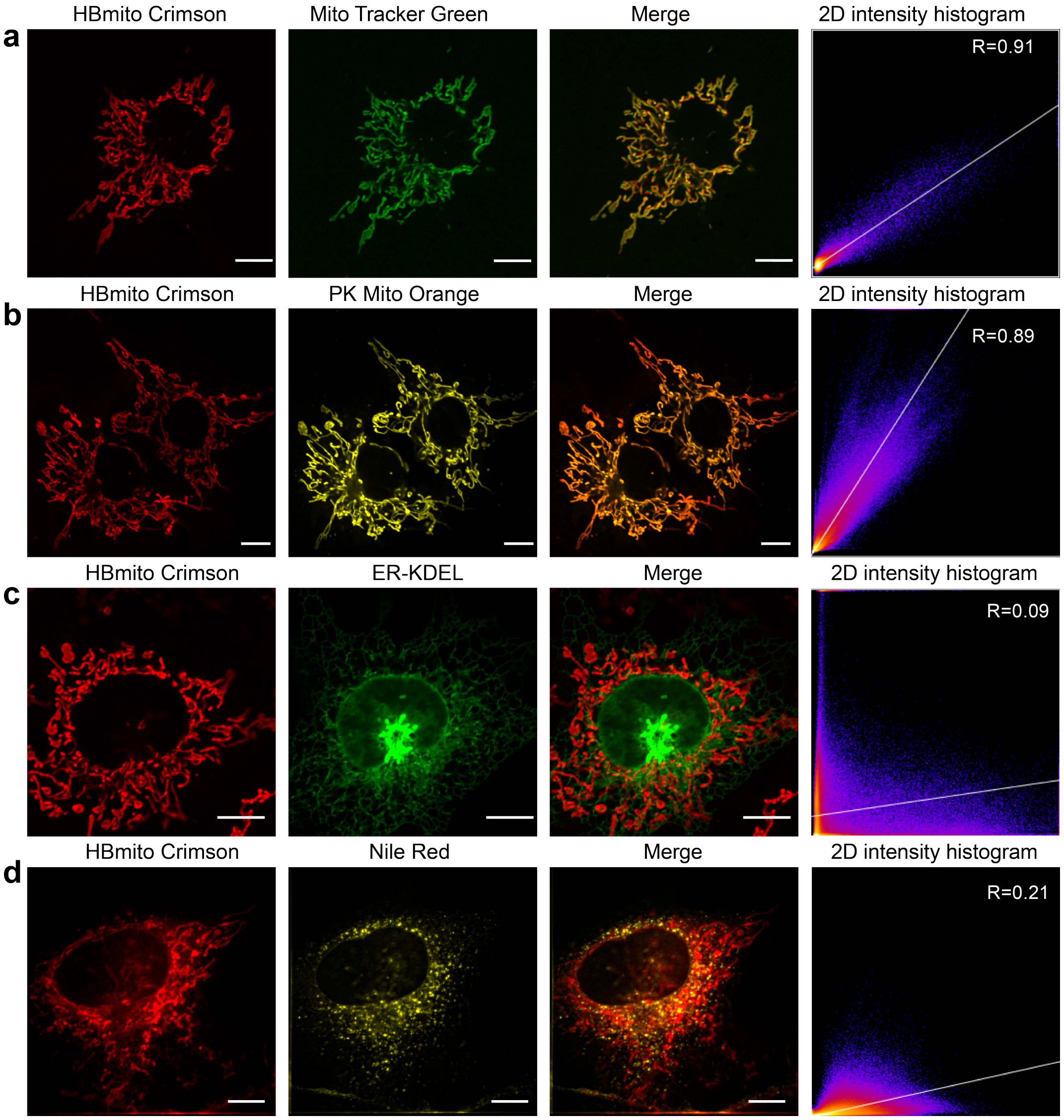

Fig. S5 Colocalization analysis of HBmito Crimson with Mito Tracker Green, PK Mito Orange, ER-KDEL and Nile Red. Scales are 10 μm.

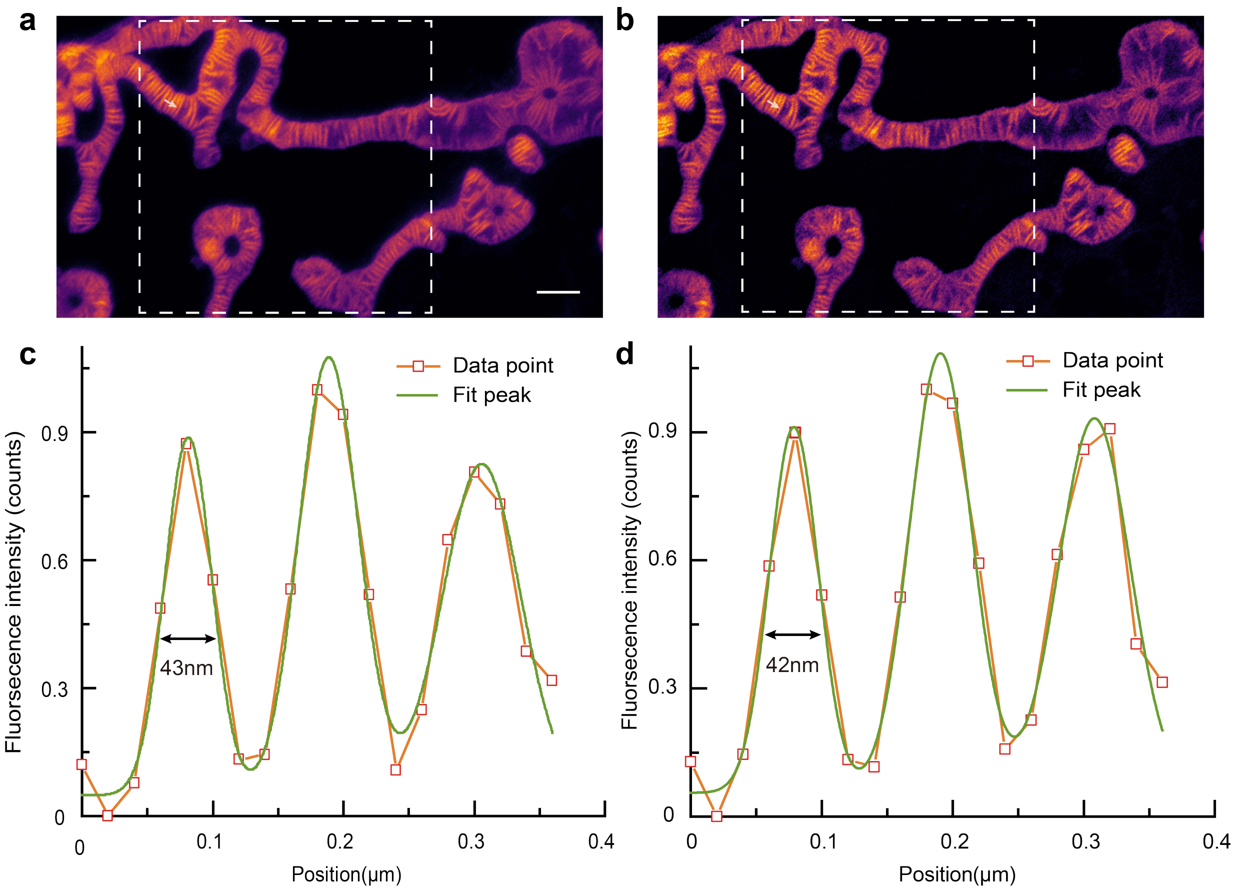

Fig. S6 STED (a) and eliminate STED secondary excitation (b) images of living COS7 cell mitochondria labeled with HBmito Crimson. (c) The signal intensity profile crossed the cristae (indicated with arrow) in Fig. S6a. (d) The signal intensity profile crossed the cristae (indicated with arrow) in Fig. S6b. Scale bar 1 μm.

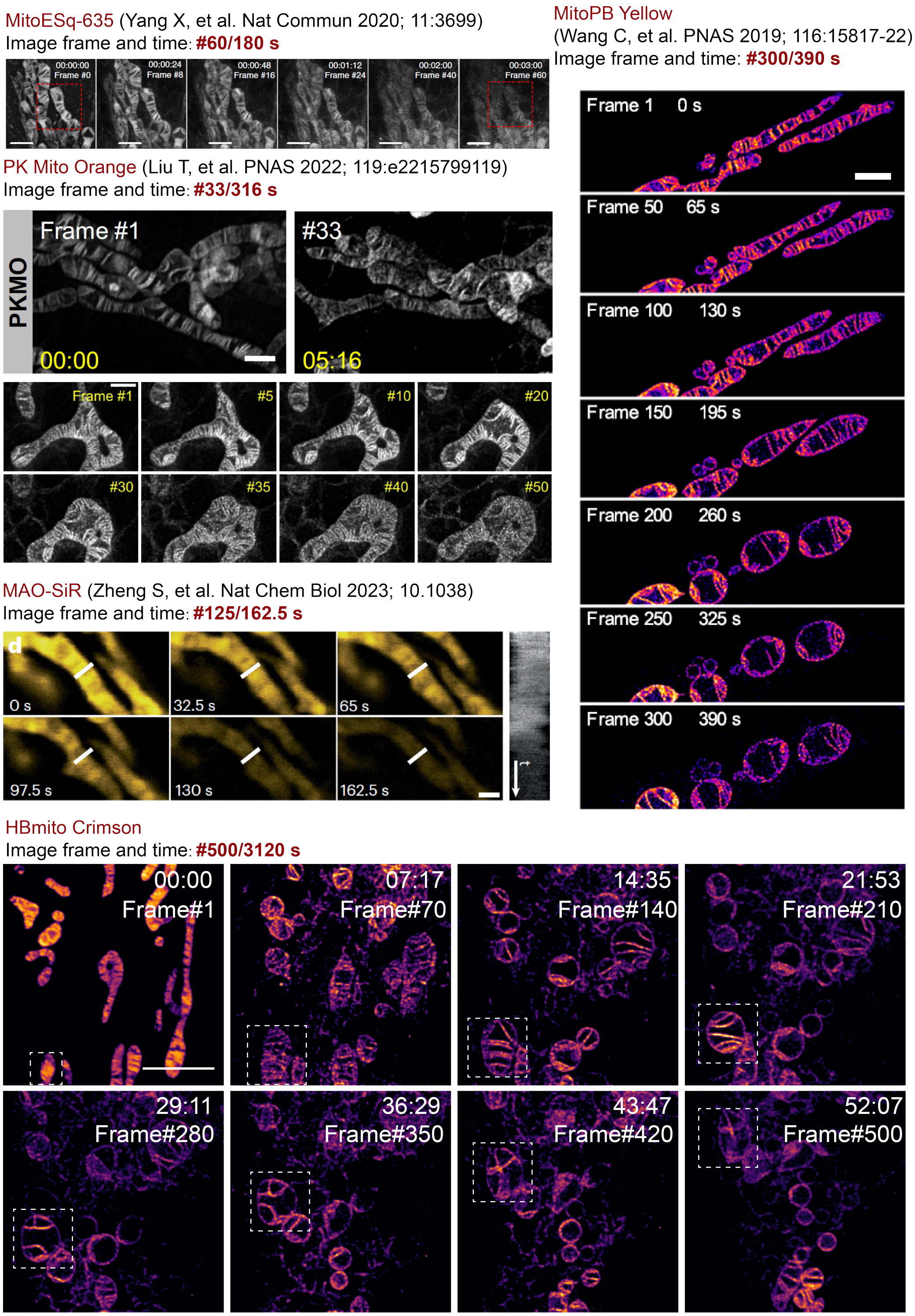

Fig. S7 Comparison of STED time-lapse imaging results of different dyes. Scale bar: MitoESq-635: 3 μm; PK Mito Orange: 1 μm; MAO-SiR: 1μm; MitoPB Yellow: 2 μm; HBmito Crimson: 3 μm.

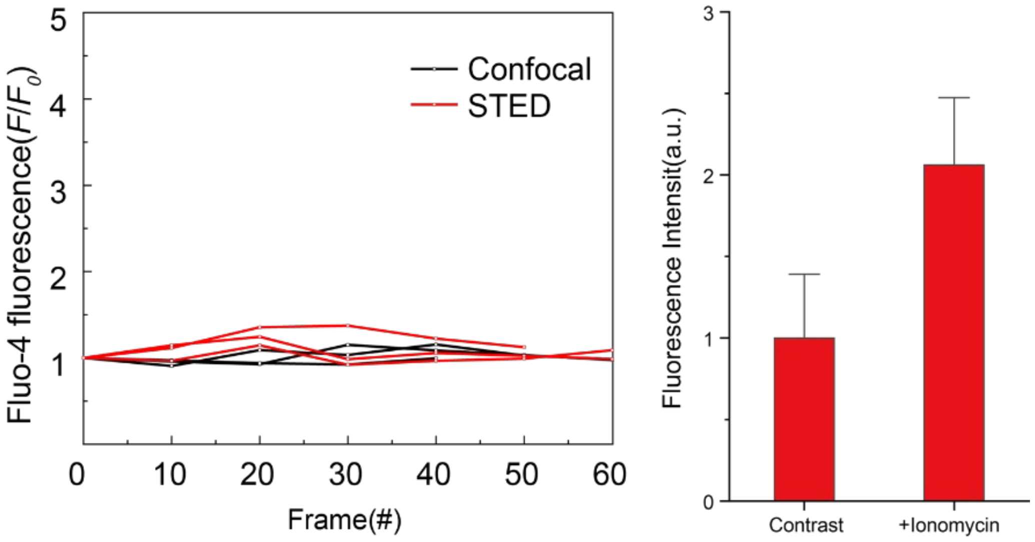

Fig. S8 (*A*) Cytoplasmic Ca^{2+}-level response of HBmito Crimson-labeled cells under confocal and STED illumination. (*B*) Results for the positive control with ionomycin (IO) treatment.

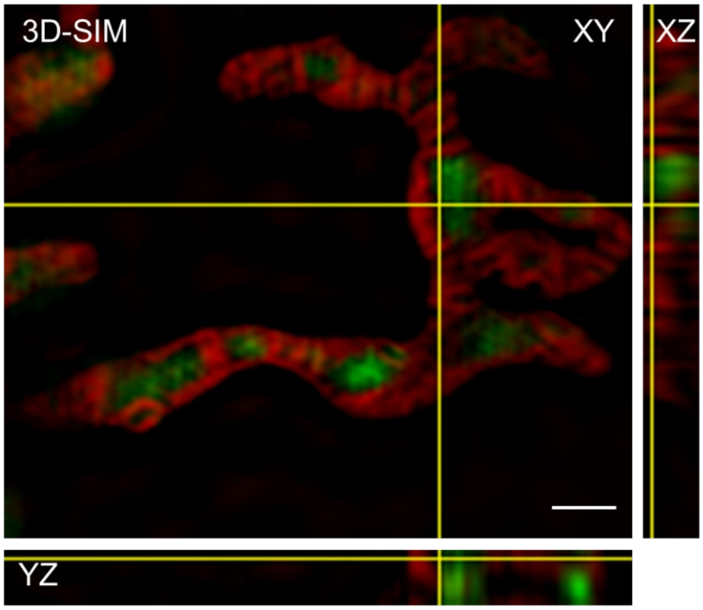

Fig. S9 The 3D-SIM results showed the distribution of mtDNA in mitochondria. Scale bar 1 μm.

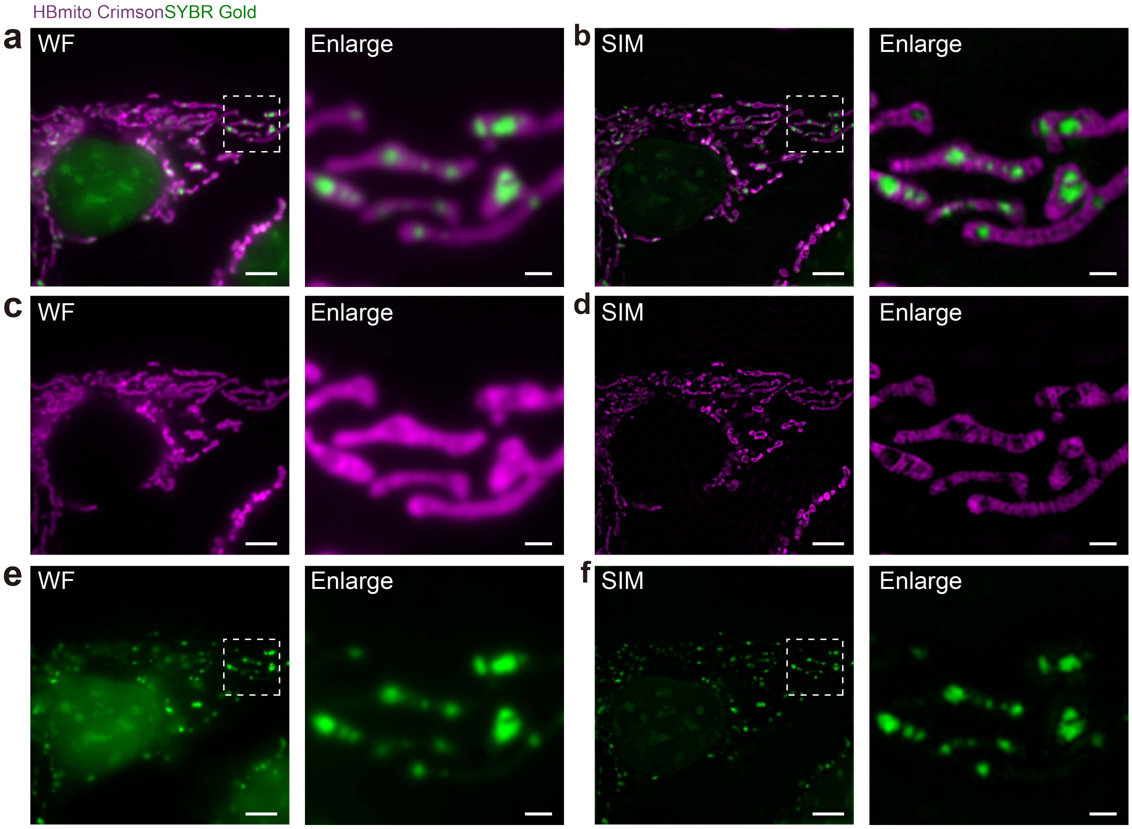

Fig. S10 Widefield and SIM results of HBmito Crimson-labeled IM and SYBR Gold-labeled mtDNA. a, b, c and d scales are 5 μm, and enlarged images are 1 μm.

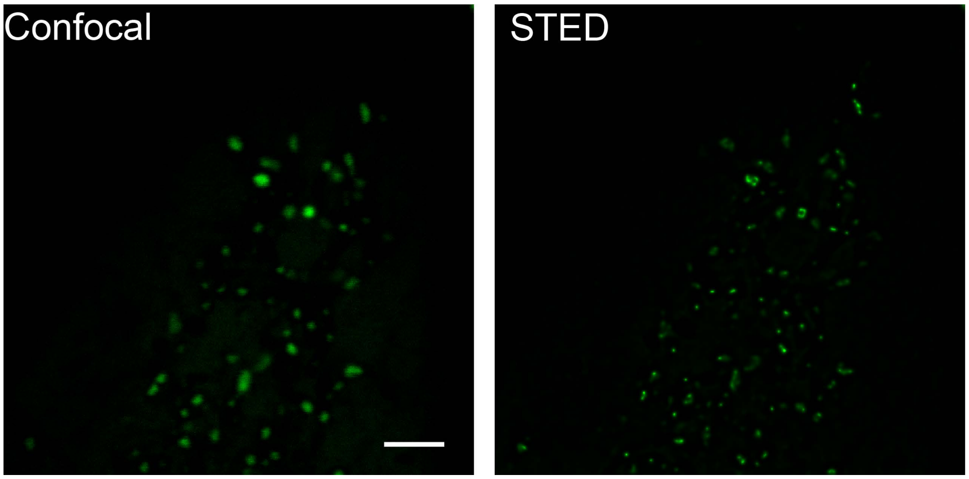

Fig. S11 Confocal and STED imaging results of SYBR Gold labeled mtDNA. Scales are 3 μm.

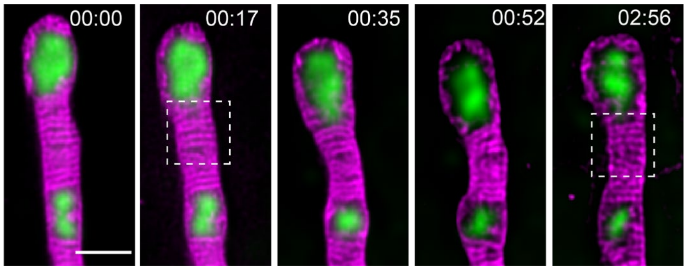

Fig. S12 Spatial changes in mtDNA and cristae at the mitochondrial tip by STED imaging. The white boxed area shows cristae remodeling. Scale bar 0.5 μm.

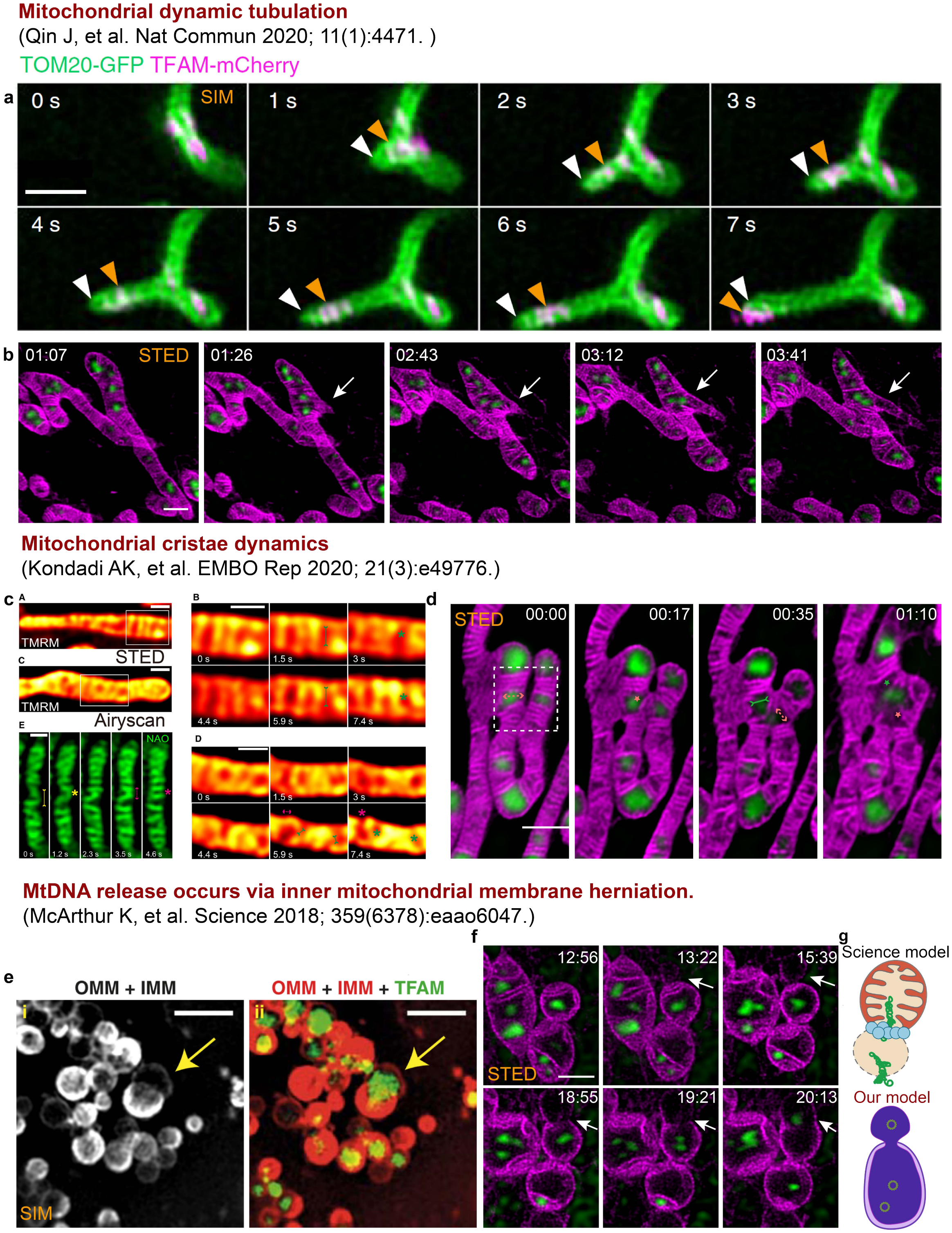

Fig. S13 Above (a, b): arrows indicate the direction of mitochondrial dynamic tubulation. Middle (c, d): bidirectional arrows facing inward and outward represent imminent cristae merging and splitting events, respectively, while an asterisk indicates completion. Bottom (e, f, g): arrows indicate the location of mitochondrial herniation. Scale bar: a: 0.5 µm; b: 1 µm; c: 2µm; d: 1 µm; f: 1µm; g: 1 µm;

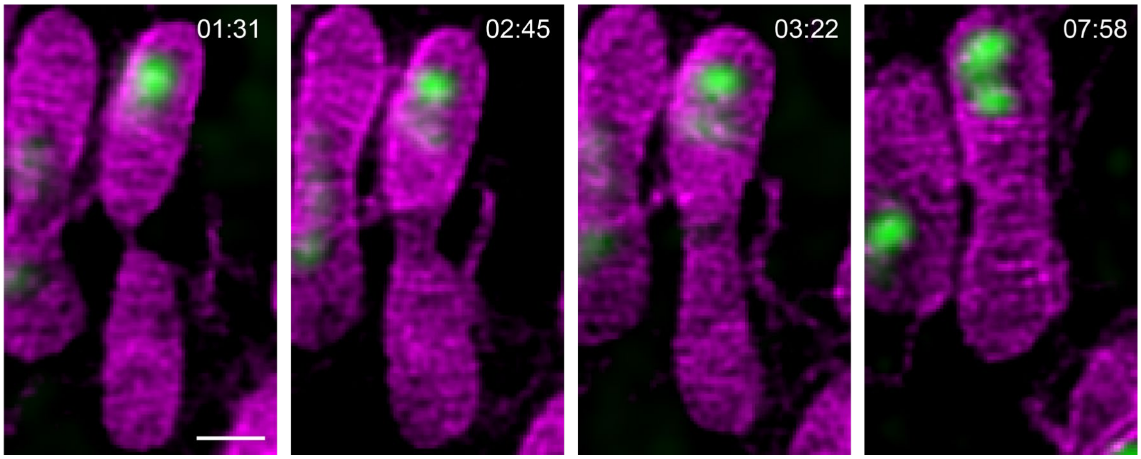

Fig. S14 Fusion of one mitochondrion containing mtDNA and another mitochondrion without mtDNA. Scale bar 0.5 µm.

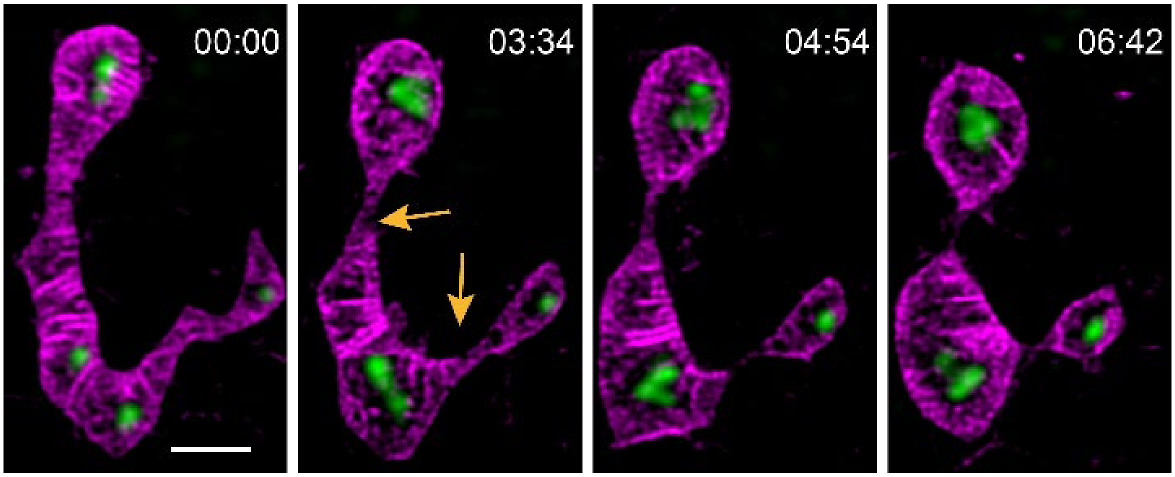

Fig. S15 Fission process of mitochondria. The yellow arrow indicates the fission site. Scale bar 1 µm.

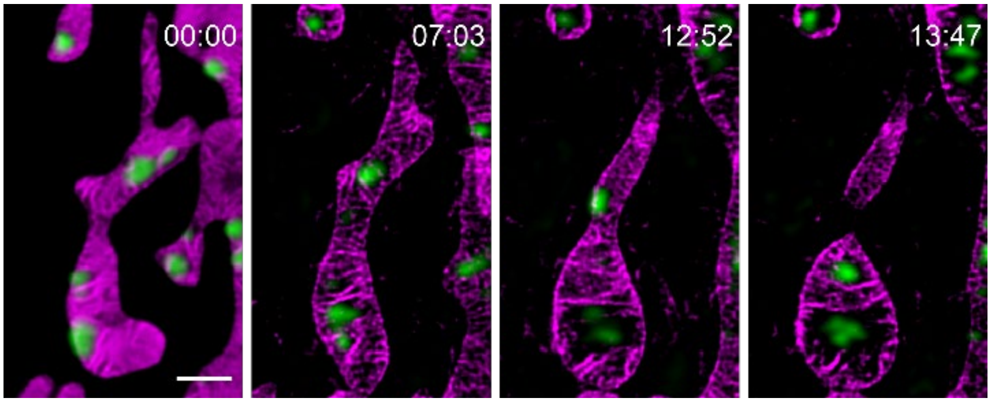

Fig. S16 Mitochondria after fission may not contain mtDNA. Scale bar 1 µm.

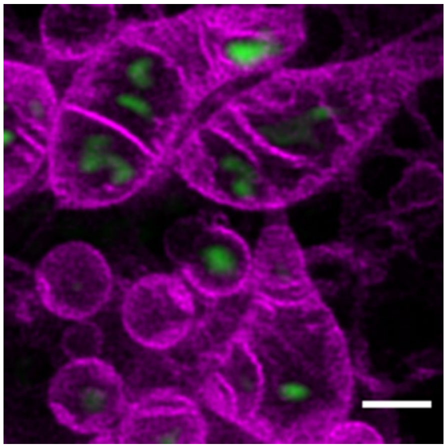

Fig. S17 Two-color STED imaging results of COS7 cells induced by ABT-737 and S63845 under STED nanoscope. Scale bar 1 μm.

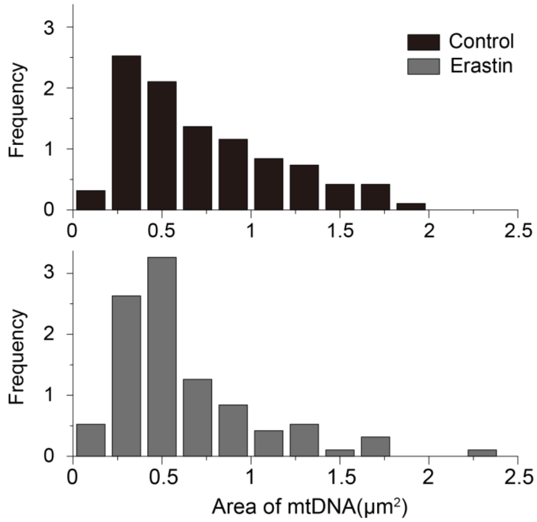

Fig. S18 Frequency distribution histogram of mtDNA area between the control and erastin-treated groups.

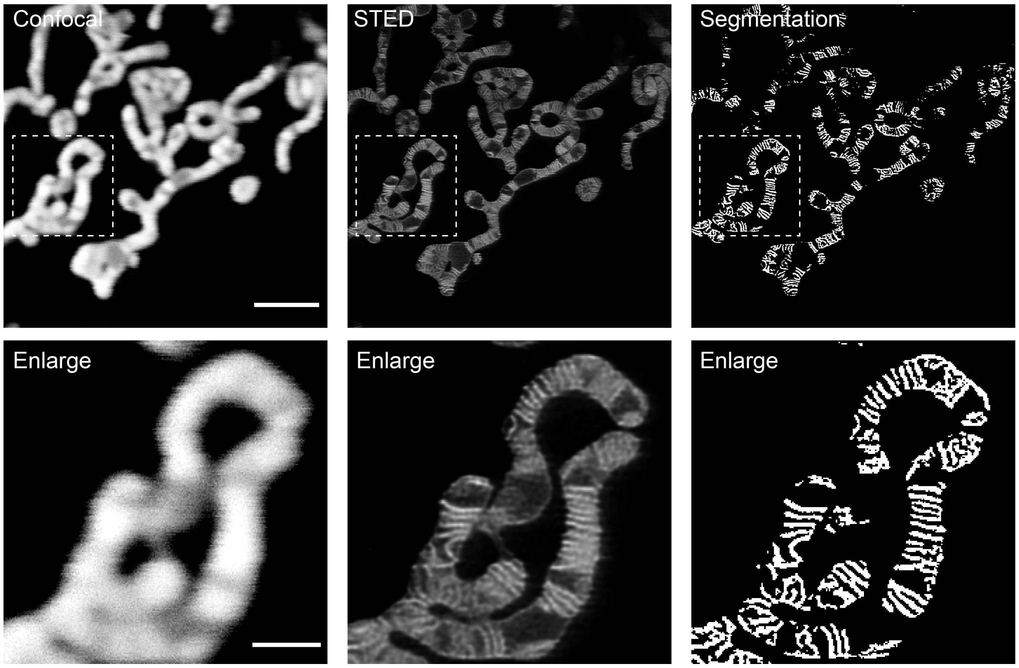

Fig. S19 Confocal, STED and STED-based mitochondria cristae segmentation results. Scales are 3 μm, and enlarged image is 1 μm.

Supplementary Tables

Table S1 Comparison of the performance of different STED probes in live cell imaging

Probes	Wavelength	Resolution (raw data)	Frame time	Field of view	Video time	Need wash
**HBmito Crimson**	Ex: 640 nm, STED: 775 nm	40 nm	6.255 s	9.2μm×9.2μm	3127.5 s, 500 frames	Wash free
**PKMO** (Liu T, et al. PNAS 2022; 119: e2215799119)	Ex: 591nm STED: 775nm	50nm	9.5s	5μm×4μm	316 s, 33 frames	yes
**MitoESq-635** (Yang X, et al. Nat Commun 2020; 11: 3699)	Ex: 635 nm, STED: 775 nm	50 nm	2.58 s	4μm×4μm	600 s, 200 frames	Wash free
**Mito PB Yellow** (Wang C, et al. PNAS 2019; 116: 15817-22)	Ex: 488 nm, STED: 660 nm	60 nm	1.3 s	5.8μm×17.4μm	390 s, 300 frames	yes
**SNAP-tag** (Stephan T, et al. Sci Rep 2019; 9: 12419)	Ex: 640 nm, STED: 775 nm	50 nm	15 s	9.6μm×12.6μm	120 s, 10-20 frames	yes

Table S2 Fluorescence (confocal and STED) microscopy data acquisition parameters.

Figure	Model	Laser setting	Objective	Pixel size	Line accumulations	Dwell time	Frames number	Frame interval
**2a**	Facility line	λ_{Ex}640nm 15.7 µW λ_{Dep}775nm 71.28 mW	60×	20nm	3	6.5µs	1	Single frame
**2b**	Facility line	λ_{Ex}640nm 14.1 µW λ_{Dep}775nm 34.7 mW	60×	30nm	2	8µs	500	6.255 s
**2c**	Facility line	λ_{Ex}640nm 12.75 µW λ_{Dep}775nm 71.28 mW	60×	25nm	2	6.5µs	13	3D
**3a**	Facility line	λ_{Ex}488 2.8 µW	60×	20nm	2	5µs	1	Single frame
		λ_{Ex}640nm 12.75 µW λ_{Dep}775nm 34.7 mW			3			
**3b**	Facility line	λ_{Ex}488 2.8 µW	60×	20nm	2	5µs	1	Single frame
		λ_{Ex}640nm 12.75 µW λ_{Dep}775nm 34.7 mW			3			
**3c**	Facility line	λ_{Ex}488 4.2 µW	60×	25nm	2	6.5µs	10	3D
		λ_{Ex}640nm 12.75 µW λ_{Dep}775nm 71.28 mW			3			
**3f**	Live-SR	λ_{Ex}488 0.4mW λ_{Ex}640nm 0.2mW	100×	54nm	1		33	0.5 s
**4b**	Facility line	λ_{Ex}488 2.8 µW	60×	20nm	1	5µs	1	Single frame
		λ_{Ex}640nm 15.7 µW λ_{Dep}775nm 71.28 mW			3			
**4c**	Facility line	λ_{Ex}488 2.8 µW	60×	25nm	1	5µs	6	12.39 s
		λ_{Ex}640nm 12.75 µW λ_{Dep}775nm 34.7 mW			3			
**4d**	Facility line	λ_{Ex}488 2.8 µW	60×	20nm	1	5µs	23	9.599 s
		λ_{Ex}640nm 12.75 µW λ_{Dep}775nm 34.7 mW			3			

**4e**	Facility line	λ_{Ex}488 2.8 µW	60×	20nm	1	5µs	7	9.599 s
		λ_{Ex}640nm 12.75 µW λ_{Dep}775nm 34.7 mW			3			
**4f**	Facility line	λ_{Ex}488 2.8 µW	60×	20nm	2	5µs	4	17.64 s
		λ_{Ex}640nm 12.75 µW λ_{Dep}775nm 34.7 mW			3			
**4i**	Live-SR	λ_{Ex}488 0.4mW λ_{Ex}640nm 0.2mW	100×	54nm	1		42	0.5 s
**5a**	Facility line	λ_{Ex}488 4.2 µW	60×	25nm	1	5µs	151	6.525 s
		λ_{Ex}640nm 12.75 µW λ_{Dep}775nm 34.7 mW			3			
**5c**	Facility line	λ_{Ex}488 4.2 µW	60×	25nm	1	5µs	187	6.525 s
		λ_{Ex}640nm 12.75 µW λ_{Dep}775nm 34.7 mW			3			
**5d**	Facility line	λ_{Ex}488 1.45 µW	60×	25nm	1	5µs	277	14.54 s
		λ_{Ex}640nm 12.75 µW λ_{Dep}775nm 34.7 mW			3			
**6a**	Facility line	λ_{Ex}640nm 15.7 µW λ_{Dep}775nm 71.28 mW	60×	20nm	3	6.5µs	1	Single frame
**6b**	Facility line	λ_{Ex}640nm 15.7 µW λ_{Dep}775nm 71.28 mW	60×	15nm	3	5µs	1	Single frame
**6e**	Facility line	λ_{Ex}488 2.8 µW	60×	25nm	1	5µs	1	Single frame
		λ_{Ex}640nm 15.7 µW λ_{Dep}775nm 71.28 mW			3			
**6f**	Facility line	λ_{Ex}488 2.8 µW	60×	20nm	2	5µs	1	Single frame
		λEx640nm 15.7 µW λDep775nm 71.28 mW			3			

Supplementary Movies

Movie S1. Time-lapse STED imaging of mitochondria labeled with HBmito Crimson. Single crista was distinctly identified and their dynamics were monitored for more than 500 frames, for a total period of 52 mins.

Movie S2. Small mitochondrial branches detach from the mitochondrial network and fuse with nearby mitochondria.

Movie S3. Branch point formation by mitochondrial fusion. During this process, the two mitochondria approach each other, and their respective mtDNA is near the fusion site. After fusion, a new branch point is established (indicated by white arrow), and since the fusion occurs near mtDNA, the mtDNA naturally resides at the branch point.

Movie S4. Branch point formation by emersion of a new branch. At the first, a new branch extends from a region where mtDNA is spatially dispersed. As the new branch grows in length and width, the mitochondrial cristae at the extension site undergo rapid remodeling to adapt to the new structure. As a result, the number of cristae at the newly formed branch point decreases, leading to a wider range of mtDNA activity, and the cristae at the new branch become relatively sparse.

Movie S5. The mtDNA on the mitochondrial branch moves to the branch point.

Movie S6. Time-lapse STED imaging of the mitochondrial fusion. During this process, two parallel mitochondria come closer together, and the IM undergoes remodeling (indicated by white box), followed by the transmission of mtDNA between them.

Movie S7. MtDNA replication initiates mitochondrial division. Initially, this long mitochondrion has only one mtDNA. At 02:15, a nascent mtDNA appeared near the original mtDNA (The dotted line shows the replication of mtDNA), then fission took place between two mtDNA, and finally, the original mitochondria were divided into two daughter mitochondria, each with one mtDNA. Meanwhile, mtDNA was located at newly generated mitochondrial tips after fission.

Movie S8. Dynamic process of cristae and mtDNA after STED time-lapse imaging.

Movie S9. IMM herniation and mtDNA leakage along with cristae remodeling. The herniated IM formed a barbell-shaped structure, and mtDNA was observed to move into the herniated IM (indicated by white arrows). During IM herniation, cristae within the mitochondria were pulled out, gradually disappeared. As the cristae within the mitochondria reduced, mtDNA was completely discharged from the tethering of mitochondrial cristae.

Movie S10. IMM herniation and mtDNA leakage.

NMR spectra of newly synthesized compounds and Mass Spectra analysis

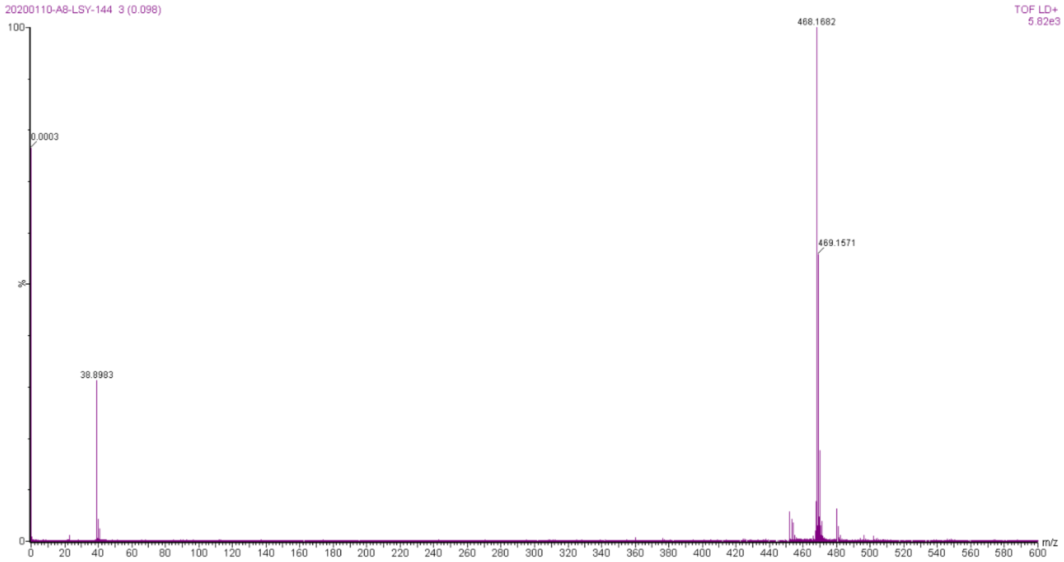

MS-1. MALDI-TOF-MS spectra of compound 3

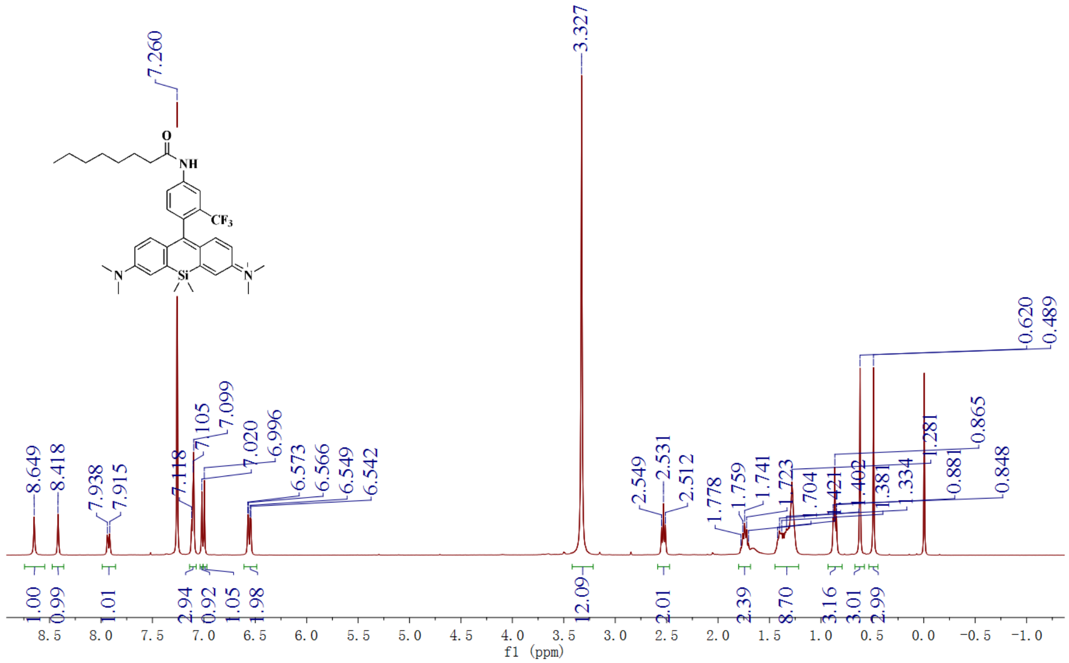

NMR-1. ^{1}H NMR (400 MHz) spectra of HBmito Crimson in $CDCl_3$.

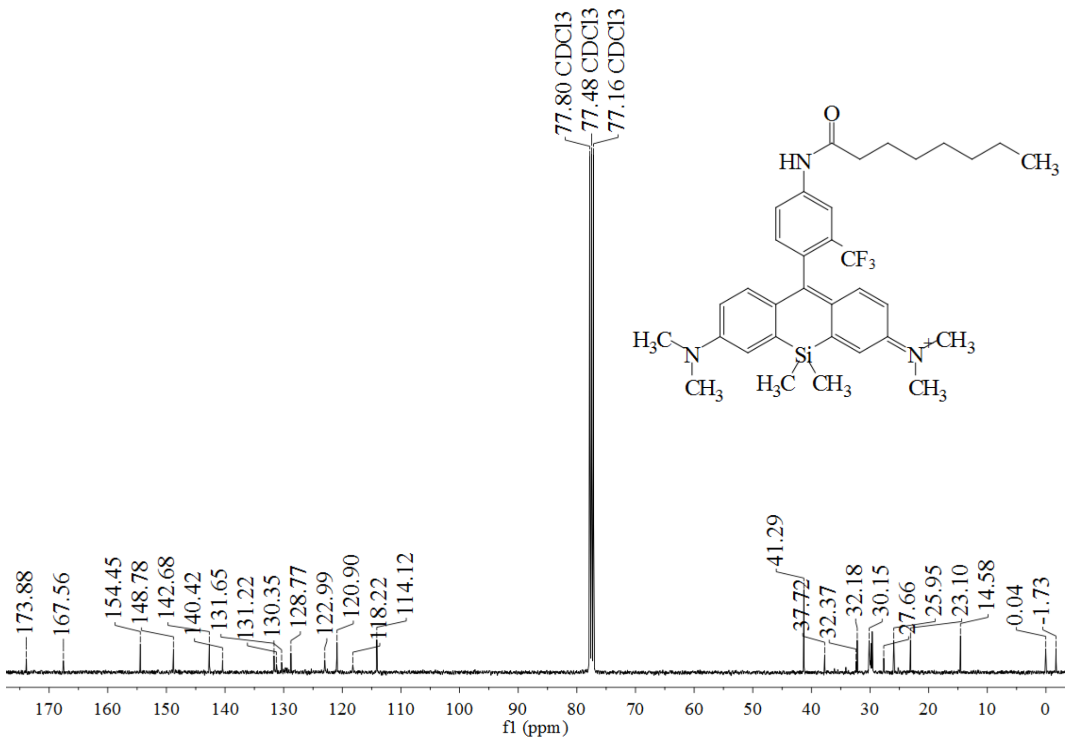

NMR-2. ^{13}C NMR (101 MHz) spectra of HBmito Crimson in $CDCl_3$.

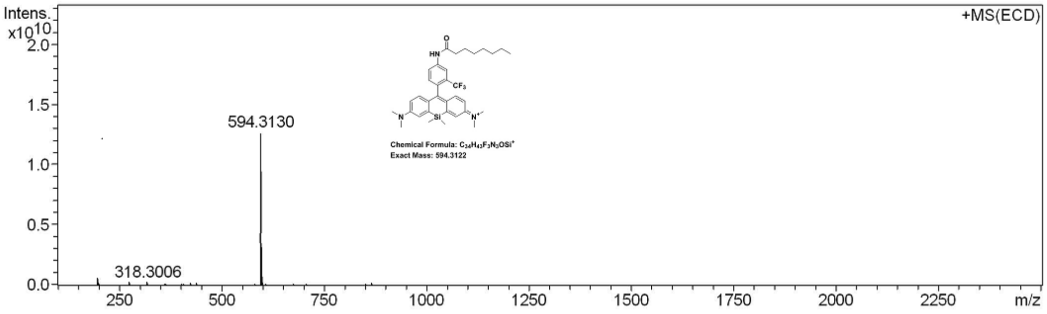

Meas. m/z	#	Formula	Score	m/z	err [mDa]	err [ppm]	mSigma	rdb	e⁻ Conf	N-Rule
594.3130	1	C 34 H 43 F 3 N 3 O Si	100.00	594.3122	-0.8	-1.4	27.3	14.5	even	ok

MS-1. High Resolution Mass Spectra of HBmito Crimson.
